# Structural basis of the transport mechanism of hBGT1

**DOI:** 10.1038/s41421-026-00926-0

**Published:** 2026-09-29

**Authors:** Kun Hao, Jiahui Chen, Jun Zhao, Renjie Li, Yue Li, Pu Yuan, Qinru Bai, Yanli Dong, Jie Yu, Yan Zhao

**Affiliations:** 1 National Laboratory of Biomacromolecules, CAS Center for Excellence in Biomacromolecules, Institute of Biophysics, Chinese Academy of Sciences, Beijing, China; 2 State Key Laboratory of Brain and Cognitive Science, Institute of Biophysics, Chinese Academy of Sciences, Beijing, China; 3 College of Life Sciences, University of Chinese Academy of Sciences, Beijing, China; 4 Ministry of Education Key Laboratory of Anesthesiology and Resuscitation, Huazhong University of Science and Technology, Wuhan, Hubei China; 5 Interdisciplinary Research Center on Biology and Chemistry, Shanghai Institute of Organic Chemistry, Chinese Academy of Sciences, Shanghai, China; 6 Peking University Institute of Advanced Agricultural Sciences, Shandong Laboratory of Advanced Agricultural Sciences at Weifang, Weifang, Shandong China; 7 School of Biological Sciences and Technology, Beijing Forestry University, Beijing, China; 8 Beijing Key Laboratory of Precision Translational Medicine in Anesthesiology and Pain, Beijing, China

**Keywords:** Cryoelectron microscopy, Molecular biology

## Abstract

In the kidney and liver, the betaine/γ-aminobutyric acid (GABA) transporter 1 (BGT1) transports betaine to maintain the osmotic balance of renal medullary cells and reduce the toxicity of homocysteine accumulation. In the brain, BGT1 reuptakes GABA from the synaptic cleft into glial cells to terminate GABAergic signaling. Despite its importance, the molecular mechanisms of BGT1 substrate recognition and ion coupling remain unknown. Here, we resolved the cryo-electron microscopy structures of BGT1 in complex with two substrates, GABA and betaine, as well as the substrate-free form without any fiducial marker. The substrate-bound complex elucidates the mechanism by which BGT1 recognizes chemically distinct GABA and betaine. These structures are trapped in occluded and inward-open states, elucidating the structural basis for conformational transitions. Furthermore, we proposed a Na3-binding site and investigated the functional role of the Na3 site in limiting reverse transport and promoting substrate accumulation. These insights improve our understanding of substrate recognition, conformational transitions, and ion coupling mechanisms in BGT1, as well as other neurotransmitter transporters.

## Introduction

The betaine/γ-aminobutyric acid (GABA) transporter 1 (BGT1) was first discovered and cloned in Madin-Darby canine kidney (MDCK) cells and subsequently identified in the liver and brain^1–4^, where it participates in various physiological processes. In renal medullary cells, BGT1 transports and accumulates betaine at high concentrations to counteract extracellular hyperosmolarity^1,5^, thereby preventing apoptosis induced by osmotic stress from high NaCl and urea concentrations^6,7^. Homocysteine is a toxic metabolite, and its accumulation in the body increases the risk of cardiovascular diseases^8–10^. In the liver, betaine is transported into hepatocytes, where it donates a methyl group to homocysteine and converts it into methionine^11–13^, which maintains homocysteine levels within the normal range to prevent the toxic effects associated with its accumulation. In the brain, BGT1 has been reported to localize to astrocytes^14^, where it likely limits the diffusion of GABA to adjacent synapses, maintaining the fidelity of synaptic signal transmission^15,16^. Furthermore, BGT1 expression is elevated in the brain during status epilepticus^17,18^. Given that BGT1-selective inhibitors have demonstrated antiepileptic effects^19,20^, BGT1 has garnered increasing attention in the development of antiepileptic drugs^21–23^.

Owing to its ability to transport GABA and sequence homology, BGT1 is classified within the GABA transporter (GAT) family, a subgroup of solute carrier family 6 (SLC6). Its activity is dependent on Na^+^ and Cl^–^^5^^,^^24^. Recently, the structures of several neurotransmitter/sodium symporter (NSS) proteins, such as GAT1^25–27^, noradrenaline transporter (NET)^28–31^, dopamine transporter (DAT)^32–35^, and serotonin transporter (SERT)^36–38^, have been resolved. However, these proteins transport their substrates with only two sodium ions, unlike BGT1, which cotransports three sodium ions^39^. This unique sodium ion coupling results in a stronger uphill transport driving force and inhibition of reverse transport^40^, which is critical for the accumulation of high concentrations of betaine in renal medullary cells. Despite its significance, the exact binding site for the third Na^+^ ion remains unclear. Moreover, unlike GAT1–3, which exclusively take up GABA, BGT1 is capable of transporting both GABA and betaine^41^. However, the molecular basis of this dual substrate selectivity remains unknown.

Here, we resolved the structures of BGT1 in the substrate-bound state and an inward-open state obtained without exogenous substrate addition during purification. These structures reveal the network of interactions between BGT1 and its substrates and ions, leading to the identification of a potential Na3-binding site. Additionally, we captured the conformational transition of BGT1 from the occluded state to the inward-open state. Our structural and biochemical characterization of BGT1 provides detailed insights into its substrate specificity and transport mechanisms.

## Results

### Structural determination and architecture of BGT1

**Fig. 1:**
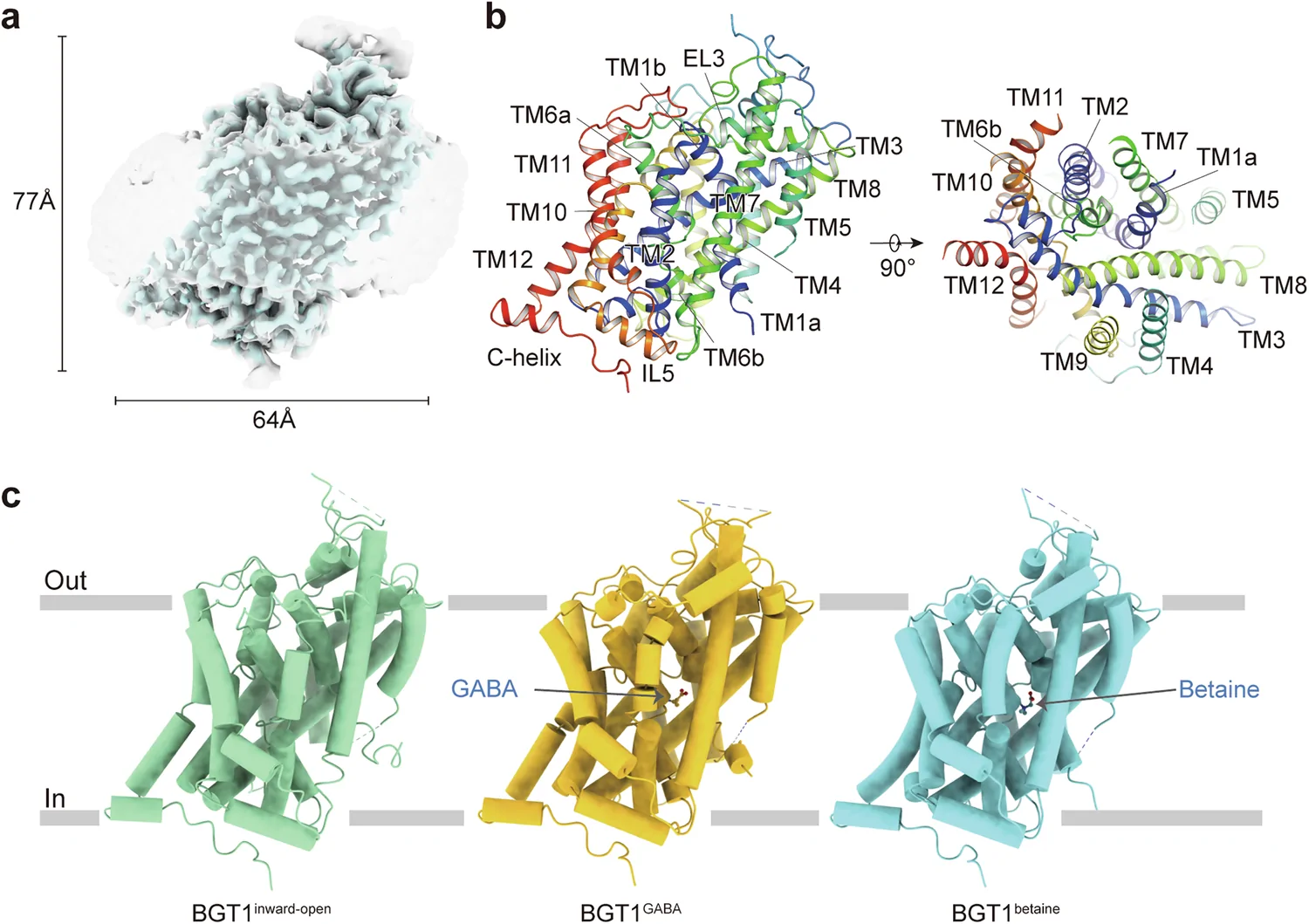
Cryo-EM structure of BGT1. **a** Cryo-EM map of nanodisc-reconstituted BGT1^betaine^ at a contour level of 7.5 σ in ChimeraX. The size of the BGT1 molecule is labeled. BGT1 and the nanodisc are represented by cyan and transparent gray, respectively. **b** Overall structure of BGT1^betaine^ with labeled transmembrane helices and intracellular and extracellular loops. The betaine molecule is not shown in the model. **c** Structures of BGT1 in the inward-open state, GABA-bound state, and betaine-bound state are colored green, gold, and cyan, respectively.

To elucidate the substrate binding and transport mechanisms of BGT1, we employed single-particle cryo-electron microscopy (cryo-EM) to determine its structure. In recent years, the structures of proteins in the SLC6 family have increasingly been resolved, often involving the use of thermostabilizing mutations or truncations to optimize their biochemical properties^28,33^. For this study, we expressed and purified full-length, wild-type (WT) BGT1 for structural determination. Specifically, we used *n*-dodecyl-β-D-maltopyranoside (DDM) to solubilize and purify BGT1, with digitonin buffer used during the size-exclusion chromatography (SEC) experiments (Supplementary Fig. S1a). The BGT1 sample was subsequently reconstituted into nanodiscs composed of brain polar lipids and MSP1D1E3 (Supplementary Fig. S1b, c). We determined the structures of BGT1 in complex with two substrates at concentrations of 10 mM GABA and 20 mM betaine, as well as an inward-open structure obtained in the absence of exogenous substrate, achieving resolutions of 2.7, 2.6, and 3.2 Å, respectively (Fig. 1a–c; Supplementary Fig. S2 and Table S1).

The overall structure of human BGT1 is approximately 77 Å long and 64 Å wide (Fig. 1a) and displays a classic LeuT-like fold^28,33,42,43^, containing 12 transmembrane helices (TMs) in which TMs 1–5 and TMs 6–10 exhibit an inverted pseudo-twofold symmetry (Fig. 1b). The helices TM1 and TM6 unwind in the middle of the transmembrane region, which is crucial for most of the interactions with the substrates and ions concentrated in this area^43^. The N- and C-termini of BGT1 are both located on the intracellular side, but owing to their disorder, we were unable to observe the N-terminus (residues 1–42) and part of the C-terminus (residues 585–614) in the cryo-EM map. However, in our structure, we observed extensive interactions between the C-terminus and the intracellular loop (IL) 1/IL5 (Supplementary Fig. S3a), which stabilized C-terminal residues (amino acids 561–584) and enabled us to resolve clear density for this region. The conserved disulfide bond in the GAT family was observed in BGT1, formed by Cys157 and Cys166 (Supplementary Fig. S3b), which stabilizes an ordered structure in part of extracellular loop (EL) 2. Two glycosylation sites, Asn171 and Asn183, are predicted on EL2^44^. However, the segment of EL2 from L170 to P180 was not observed, nor were glycosylations detected, likely due to their flexibility. In the inward-open state, BGT1 (BGT1^inward-open^) creates a pathway from the intracellular membrane surface to the substrate-binding pocket. Despite the absence of exogenously added substrates during purification, residual density was observed within the substrate-binding pocket, which may originate from a small amount of endogenously bound GABA derived from HEK293F cells^27^. We also observed density at the conserved chloride ion-binding site in the BGT1^inward-open^ structure, suggesting that the chloride ion contributes to the structural stability (Supplementary Fig. S3c). Additionally, we identified one binding site for cholesterol and one for cholesteryl hemisuccinate (CHS) in the BGT1^inward-open^ structure: one situated between TM3 and TM4 and the other within a groove formed by TM9, TM10, and TM12.

### Substrate binding and selectivity of BGT1

**Fig. 2:**
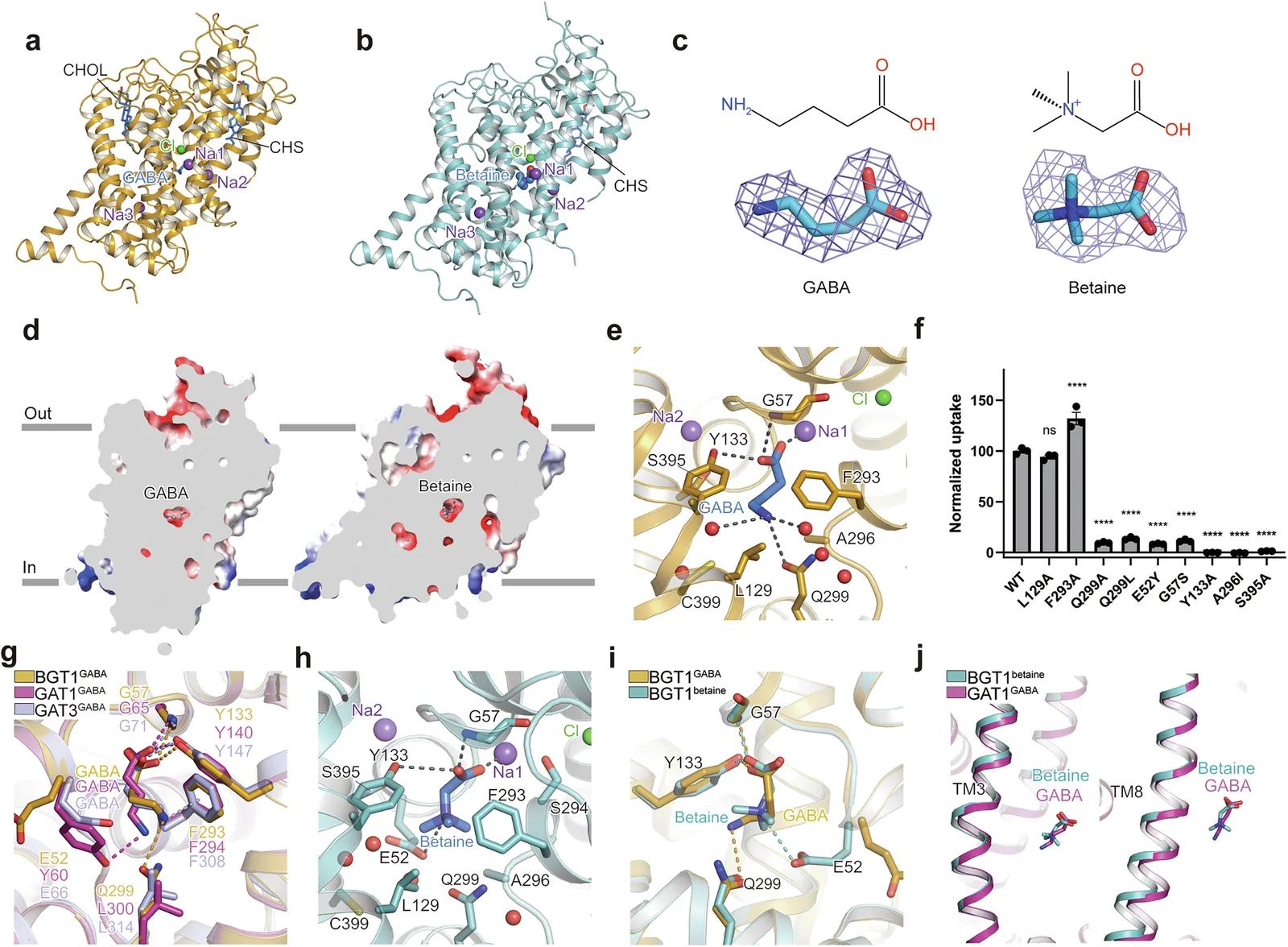
Substrate binding of BGT1. **a**, **b** Overall structures of BGT1 in complex with GABA (**a**) and betaine (**b**). Substrates, Na^+^ and Cl^‒^ are depicted as blue, purple and green spheres, respectively. **c** Chemical structure (top) and cryo-EM density (bottom) of GABA and betaine contoured at 4 σ in PyMOL. **d** Cross section of the electrostatic surface of BGT1; the substrates are shown as sticks and spheres. **e** Substrate binding pockets of BGT1^GABA^. Residues involved in GABA coordination are shown as sticks and labeled. Interactions are presented with black dashes. Na^+^, Cl^‒^ and water molecules are depicted as purple, green and red spheres, respectively. **f** The transport activities of WT BGT1 and its mutants related to substrate binding were quantified using [^3^H]-labeled GABA uptake assays. The transport activities of the WT and each mutant were adjusted based on their expression levels. The transport activity of each mutant was normalized to that of the WT. A 3-min incubation was performed to ensure that measurements were taken within the linear range. The data represent the means ± SEM (error bars); *n* = 3 biologically independent experiments. ****P < 0.0001; NS not significant. **g** Comparison of binding pockets among BGT1^GABA^, GAT1^GABA^ (PDB: 7Y7W) and GAT3^GABA^ (PDB: 9QO9). The structures of BGT1^GABA^, GAT1^GABA^ and GAT3^GABA^ are colored gold, purple and light blue, respectively. Vital residues and interactions are shown. **h** Substrate-binding pockets of BGT1^betaine^. The representation style is the same as that in **e**. **i** Superposition of the GABA-binding pockets of BGT1^GABA^ and BGT1^betaine^ colored gold and cyan, respectively. Key residues involved in GABA binding are shown as sticks and labeled. **j** Comparison of TM3 (left) and TM8 (right) between BGT1^betaine^ and GAT1^GABA^ colored cyan and purple. Helices and ligands are labeled.

To elucidate the substrate-binding mode of BGT1, we determined the structures of BGT1 bound to GABA (BGT1^GABA^) and betaine (BGT1^betaine^) (Fig. 2a, b). In our maps, we found the density that could accommodate the substrate (Fig. 2c). The substrate is in the mid-region of BGT1, equidistant from the intracellular and extracellular surfaces of the membrane. The binding pocket is inaccessible from both the extracellular and intracellular sides (Fig. 2d), indicating that the BGT1^GABA^ structure adopts an occluded conformation. The substrate-binding pocket is surrounded by TM1, TM3, TM6, and TM8, with residues G57 in TM1 (G57^TM1^), Y133^TM3^, S294^TM6^, A296^TM6^, Q299^TM6^, and S395^TM8^ maintaining a stable substrate-binding environment. Specifically, the backbone nitrogen of G57^TM1^ and the hydroxyl oxygen of Y133^TM3^ form hydrogen bonds with the carbonyl oxygen of GABA. The side-chain carbonyl oxygen of Q299^TM6^ forms a hydrogen bond with the amino nitrogen of GABA (Fig. 2e). Additionally, five water molecules identified near GABA in the structure participate in a complex hydrogen bond network with the amino end of the substrate and residues Q299^TM6^, F293^TM6^, I297^TM6^, S395^TM8^ and Q396^TM8^ (Fig. 2e; Supplementary Fig. S4a). These water molecules likely play a critical role in substrate binding in a mechanism similar to those observed in other transporters, such as DAT, GlyT1, and GAT1^26,33,43^. Hydrophobic residues, including L129^TM3^, F293^TM6^ and A296^TM6^, together with polar residues, including S294^TM6^, S395^TM8^ and C399^TM8^, stabilize the binding pocket (Fig. 2e). [^3^H]GABA uptake assays revealed that the G57S and Q299A mutations led to a significant reduction in GABA transport activity in BGT1 and that the Y133A, A296I and S395A mutations caused the loss of transport activity, although these mutant proteins were delivered to the plasma membrane (Fig. 2f; Supplementary Fig. S5). Conversely, replacements such as L129A and F293A had little impact on transport activity (Fig. 2f). These results indicate that the binding of the substrate is predominantly mediated by hydrogen bonds, while hydrophobic residues create a suitable hydrophobic environment and provide sufficient space to accommodate the substrate. A comparison with the GABA-bound structure of GAT1^26^ and GAT3^45^ revealed that interactions at the carboxyl of GABA are conserved. In contrast, recognition of the GABA amino group differed substantially among the three subtypes. In BGT1, Q299^TM6^ forms a hydrogen bond with the amino nitrogen of GABA. This interaction appears to be specific to BGT1, as the equivalent position is occupied by leucine in both GAT1 and GAT3. In GAT1, the amino group adopts a different orientation and forms a hydrogen bond with Y60^TM1^, a residue unique to GAT1 that is replaced by glutamate in BGT1 and GAT3. In GAT3, the amino group is positioned close to the phenyl ring of F308^TM6^ and is stabilized through a cation–π interaction. Although the corresponding phenylalanine is conserved in BGT1 and GAT1, it does not interact with GABA in those structures. Together, these observations reveal that the recognition of the GABA carboxylate group is conserved, whereas the recognition of the amino group is subtype-specific (Fig. 2g).

Betaine is the other substrate of BGT1. The binding pocket for betaine is highly similar to that for GABA, with the interactions of the carboxyl group being fully conserved. Like GABA, betaine interacts with residues Y133^TM3^, G57^TM1^, and Na1. Unlike the amino group of GABA, betaine has a positively charged quaternary ammonium, and its nitrogen atom forms a salt bridge with the carboxyl group of E52 (Fig. 2h, i). In the betaine-binding pocket, we also identified several water molecules according to the cryo-EM map, which are positioned differently from those involved in GABA coordination in the BGT1^GABA^ structure. These water molecules form a dense hydrogen bond network with residues E52^TM1^, N130^TM3^, I297^TM6^, Q299^TM6^, C301^TM6^, S395^TM8^ and Q396^TM8^ (Fig. 2h; Supplementary Fig. S4b). These interactions coordinate the helices TM1, TM3, TM6, and TM8 and stabilize the integrity of the betaine-binding pocket.

BGT1 is capable of transporting both GABA and betaine, whereas GAT1–3 only transport GABA^41^. To understand the molecular mechanism underlying this substrate specificity, we conducted a sequence alignment and discovered that the Q299 residue in BGT1 (Q299^BGT1^) corresponds to a leucine in GAT1–3 (Supplementary Fig. S6). This substitution with a hydrophobic side chain may hinder the formation of a suitable binding pocket for betaine in GAT1–3, thus limiting their ability to transport betaine. A comparison with the structure of GAT1 revealed a slight outward tilt of TM3 and TM8 in BGT1, which may increase the accommodation of the trimethylammonium group of betaine (Fig. 2j). Furthermore, we performed sequence alignment and compared the binding pockets of BGT1 with those of other NSS family proteins (Supplementary Fig. S7a–e). For instance, GlyT1, which transports glycine, shows highly conserved interactions with the carboxyl group of the substrate^43^ (Supplementary Fig. S7b). However, Q299^BGT1^ is substituted by W322^GlyT1^. Its large side chains restrict the size of the substrate-binding pocket, facilitating the binding of glycine, which has a shorter carbon chain^43^ (Supplementary Fig. S7b). Sequence alignment further revealed that the other glycine transporter, GlyT2, also contains a tryptophan residue at the corresponding position, indicating that this feature is conserved among glycine transporters (Supplementary Fig. S7a). With respect to monoamine transporters, such as SERT^46^, DAT^33^, and NET^28^, the amino groups of monoamines are oriented toward the extracellular region, which differs significantly from those of GABA in BGT1, and are stabilized by electrostatic interactions from aspartate at the same position as G55^BGT1^ (Supplementary Fig. S7c–e). Additionally, unlike Q299^BGT1^, which stabilizes the binding of GABA, monoamines are coordinated by F341^SERT^, F326^DAT^, and F323^NET^. In summary, the residue Q299^BGT1^ varies systematically across the NSS protein family and is correlated with variations in substrate (Supplementary Fig. S7a), suggesting that this residue may contribute to substrate selectivity among NSS transporters.

### Ion binding of BGT1

**Fig. 3:**
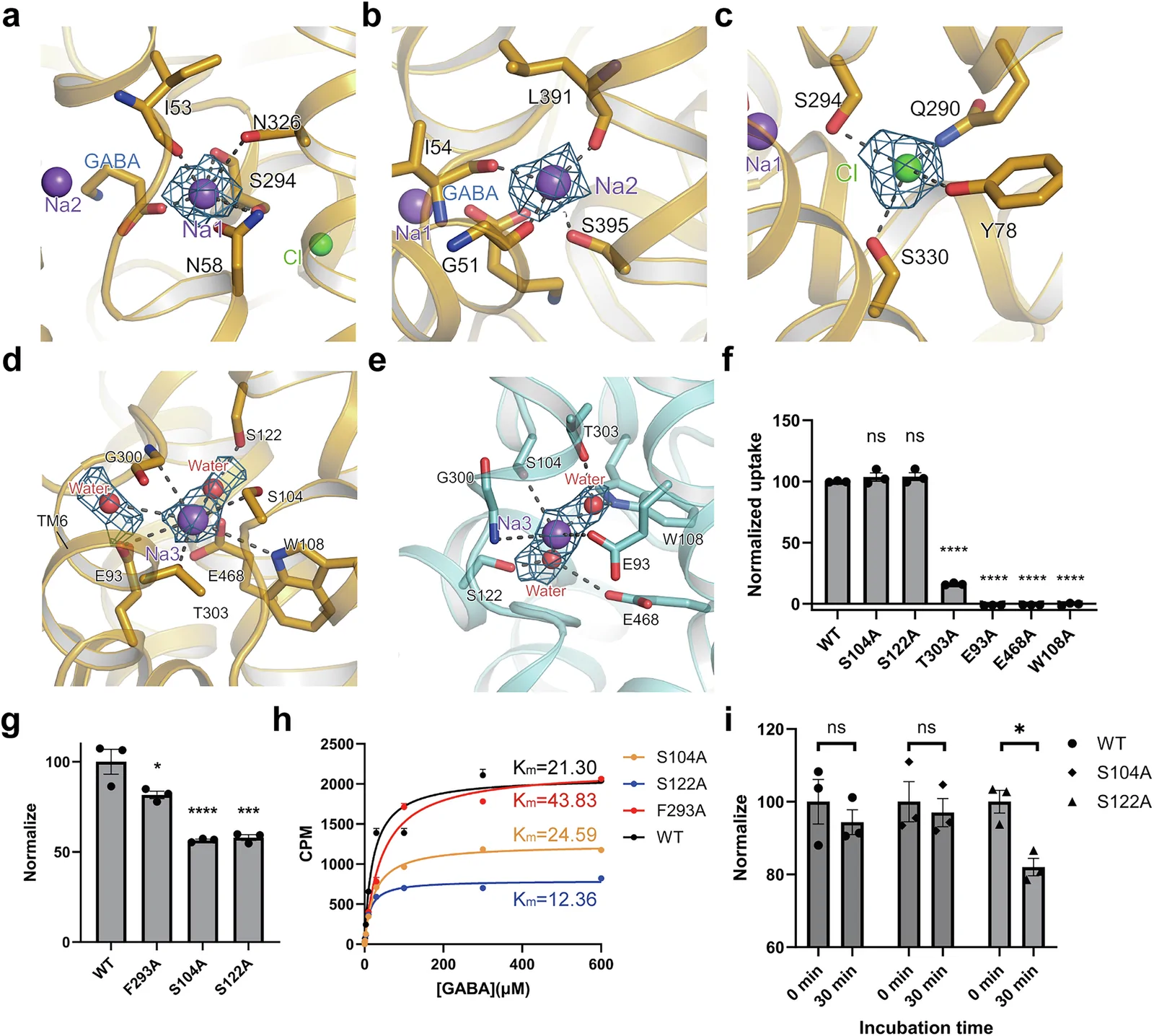
Ion-binding sites of BGT1 and functional analysis of the putative Na3 site. **a**–**c** Binding sites for Na1 (**a**), Na2 (**b**), and Cl^‒^ (**c**). Residues involved in interactions are shown as sticks and labeled. The coordination of ions is indicated with black dashes. The cryo-EM density is contoured at 4 σ in PyMOL. **d**, **e** Na3 sites in the structures of BGT1^GABA^ (**d**) and BGT1^betaine^ (**e**). Water molecules are shown as red spheres. The residues and helices involved in the interaction network are labeled. The cryo-EM density is contoured at 4 *σ* in PyMOL. **f** The transport activities of WT BGT1 and its mutants related to Na3 binding were quantified using [^3^H]-labeled GABA uptake assays. The transport activities of the WT and each mutant were adjusted based on their expression levels. The transport activity of each mutant was normalized to that of the WT. A 3-min incubation was performed to ensure that measurements were taken within the linear range. The data represent the means ± SEM (error bars); *n* = 3 biologically independent experiments. ****P < 0.0001; NS not significant. **g** Saturated [^3^H]-labeled GABA uptake measured after 2 h of transport. The data were adjusted according to the cell expression levels. The mutation data were normalized to the WT data. The data represent the means ± SEM (error bars); *n* = 3 biologically independent experiments. *P < 0.05, ***P < 0.001, ****P < 0.0001. **h** Concentration-dependent substrate uptake kinetic curves for WT (black), F293A (red), S122A (blue), and S104A (orange) of BGT1. The data were obtained from three biologically independent replicate experiments. The data represent the means ± SEM (error bars); *n* = 3 biologically independent experiments. The *K*_m_ values of WT, F293A, S122A, and S104A were 21.30 ± 1.27, 43.83 ± 3.47, 12.36 ± 1.09, and 24.59 ± 1.13 μM, respectively. There was a significant difference between the *K*_m_ values of WT and F293A (*P* = 0.0035), whereas the differences between WT and S122A (*P* = 0.1204) and S104A (*P* = 0.2800) were not significant. Statistical significance was determined using unpaired *t*-tests with α = 0.05. **i** Reverse transport of WT (dots) and mutant S104A (squares), and S122A (triangles). Incubation time refers to the time that cells that had transported [^3^H]-labeled GABA for 3 min were incubated in substrate-free buffer after being washed. The 30-min data were normalized to the corresponding 0 min data. The data represent the means ± SEM (error bars); *n* = 3 biologically independent experiments. In cells expressing WT BGT1 and the S104A mutant, the substrate concentration did not significantly change after a 30 min incubation period (*P* = 0.4682 for WT and *P* = 0.6817 for S104A). In contrast, cells expressing the S122A mutant exhibited a significant reduction in intracellular [³H]GABA levels after incubation (*P* = 0.0102). Statistical significance was determined using unpaired *t*-tests with *α* = 0.05. *P < 0.05; NS not significant.

NSS transporters typically utilize one chloride ion and two sodium ions to transport their substrates across the membrane^24^. However, previous investigations have reported that BGT1 requires three sodium ions for substrate transport^39^. In the resolved structures, the ion binding sites are clearly defined. Both Na1 and Na2 are situated near TM1b, with Na1 interacting with the substrate, I53, S294, and N326 (Fig. 3a), while Na2 interacts with I54, L391, S395, and G51 (Fig. 3b). G51, I53, and I54 are located on TM1a, which is closely associated with conformational transformations, suggesting that the binding of Na1 and Na2 may be related to these conformational changes. The chloride ion is positioned near TM6 and TM7, coordinating with residues S294, Q290, Y78, and S330 (Fig. 3c). The binding sites for Na1, Na2, and Cl^–^ are conserved, as observed in other NSS transporters^33,43^.

In our high-resolution cryo-EM maps of BGT1^GABA^ and BGT1^betaine^, a sphere-shaped density is observed between TM2, TM3, and TM6 near the unwound region of TM6. We speculate that this may represent the Na3 site. In the structure of BGT1^GABA^, Na3 directly interacts with the side-chain hydroxyl oxygens of S104 and T303, the indole nitrogen of W108, the backbone nitrogen of G300, and the carboxyl oxygen of E93. It also coordinates indirectly with S122 and E468 through two water molecules (Fig. 3d). In the BGT1^betaine^ structure, Na3 engages in interactions that are largely similar to those observed in BGT1^GABA^, with the involvement of two water molecules (Fig. 3e). Notably, T303 interacts with Na3 indirectly via a water-mediated contact. These coordinations and the negative electrostatic contribution from E93 and E468 collectively create an environment favorable for sodium ion binding. A local structural comparison of the Na3 site in BGT1 with that in hGAT1^26^ and hGlyT1^43^, which lack a Na3 site, reveals that this region in BGT1 is more compact due to Na3 binding (Supplementary Fig. S8a, b). Among the residues involved in these interactions, E93, W108, and E468 are highly conserved within the SLC6 family, whereas S104 and S122 are not conserved in other transporters (Supplementary Fig. S8c). [^3^H]GABA uptake assays revealed that E93A, W108A, and E468A point mutations resulted in the loss of BGT1 transport activity and T303A significantly reduced the transport activity, whereas the S104A and S122A mutants still retained GABA transport activity (Fig. 3f).

The S104A and S122A mutants were assayed for the sodium dependence of GABA transport using choline as an alternative cation and Na^+^ concentrations ranging from 0–300 mM. The apparent Na^+^ EC_50_ values increased from 74.7 ± 3.9 mM for WT BGT1 to 104.7 ± 1.1 mM and 102.2 ± 2.7 mM for S104A and S122A, respectively, indicating reduced Na^+^ affinity for BGT1 (Supplementary Fig. S8d). These results suggest that these two residues are critical for the Na3 site. Previous studies have shown that the Na3-binding site prevents reverse transport and helps accumulate higher levels of substrate within the cell, thereby supporting metabolic or osmotic needs^47^. To further validate the Na3-binding site, we assessed the saturated substrate uptake kinetics and the impact of reverse transport by introducing S104A and S122A mutations. We employed the substrate-binding pocket mutant F293A as a control to determine whether the impact of the Na3 mutation on the saturation transport capacity is specific. Our results demonstrated that Na3 mutation significantly reduced intracellular GABA levels after 2 h of saturated transport and that the substrate could not accumulate to similar concentrations as those observed in cells with WT BGT1. We also observed a modest decrease in the saturation transport of F293A, which we attributed to its impact on substrate affinity (Fig. 3g). Consequently, we determined the kinetic curves for the WT and three mutants. The results confirmed our hypothesis: the *K*_m_ of F293A was greater than that of the WT, whereas the Na3 mutation did not have such an effect (Fig. 3h). Additionally, to monitor reverse transport, cells that had accumulated [^3^H]GABA for 3 min were transferred to a GABA-free buffer. No significant reverse transport was observed within 30 min for the WT BGT1 or the S104A mutant (Fig. 3i), whereas cells expressing the S122A mutant showed a significant decrease in the intracellular GABA concentration, suggesting that residues S104 and S122 may play nonequivalent roles. These findings support the idea that this site may serve as the third Na^+^-binding site.

### Intracellular and extracellular gates

**Fig. 4:**
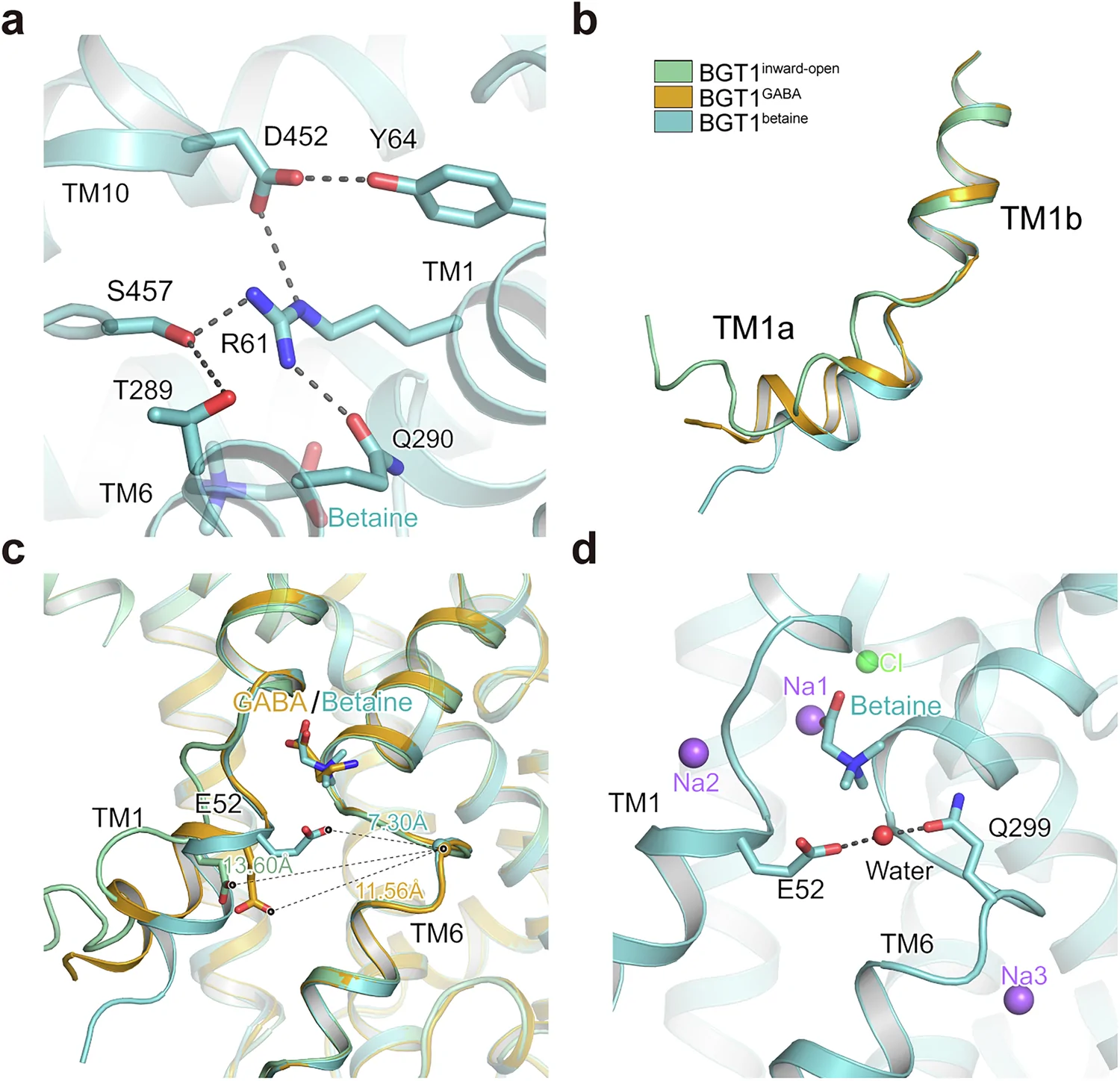
Conformational transition of BGT1. **a** Interaction network blocking the extracellular pathway, using the structure of BGT1^betaine^ as an example. The residues involved are represented as sticks. **b** Comparison of TM1 among BGT1^inward-open^, BGT1^GABA^ and BGT1^betaine^ colored green, gold and cyan, respectively. **c** Structural alignment of BGT1^inward-open^, BGT1^GABA^ and BGT1^betaine^, with TM1 and TM6 highlighted. The distances between the oxygen atom of E52 and the C_α_ of Q299 are labeled. **d** Interactions among E52, Q299 and H_2_O in the structure of BGT1^betaine^. Hydrogen bonds are depicted as black dashes. The betaine and ions are labeled.

The structure of BGT1^inward-open^ clearly delineates a pathway that allows substrate access to the central binding pocket from the intracellular side, indicating that the structure adopts an inward-open conformation. In contrast, the substrate-bound structures assume an occluded state, as previously discussed. Although these three structures display distinct conformations, the pathway from the extracellular surface to the substrate-binding pocket is obstructed and stabilized by interactions involving residues from TMs 1, 6, and 10, specifically R61^TM1^, Y64^TM1^, T289^TM6^, Q290^TM6^, D452^TM10^ and S457^TM10^ (Fig. 4a). The residues R61 and D452, which are conserved across other GABA transporters (Supplementary Fig. S6), play crucial roles in these interactions, which are essential for transport activity^43^. Unlike the nearly identical structure of the extracellular half in these states, the intracellular half of the structure exhibits significant conformational changes. Specifically, we observed that the angle between TM1a and TM6b progressively increases from the betaine-bound state to the GABA-bound state to the inward-open state (Fig. 4b, c), highlighting a transition from an occluded state to an inward-open state. In the betaine-bound state, E52^TM1^ forms an electrostatic interaction with the quaternary amine of betaine and is located within the substrate-binding pocket, establishing hydrogen bonds with Q299^TM6^ and blocking access to the intracellular surface (Fig. 4d). From the betaine-bound state to the GABA-bound state to the inward-open state, E52^TM1a^ gradually shifts away from TM6, leading to the loss of the aforementioned hydrogen bonds in both the BGT1^GABA^ and BGT1^inward-open^ structures. The side chains of E52 and C301 still obstruct the intracellular pathway in the BGT1^GABA^ structure. Moreover, during the shift from the occluded state to the inward-open state, the helix of TM1a becomes unwound (Fig. 4b). These observations enhance our understanding of the conformational transition in BGT1.

### Cholesterol-binding site

During the purification of BGT1, the buffer was supplemented with CHS, a cholesterol derivative. Based on cryo-EM density maps, we identified one CHS-binding site and one cholesterol-binding site located within the extracellular transmembrane region. These may represent the cholesterol-binding sites in the cell membrane, as CHS resembles cholesterol. The CHS-binding site, CHOL1, is between TM3 and TM4 (Supplementary Fig. S9a), and the cholesterol site, CHOL2, is found in a groove formed by TM9, TM10, and TM12 (Supplementary Fig. S9b). At the CHOL1 site, CHS coordinates with F145^TM3^, L215^TM4^, L216^TM4^, L218^TM4^, L219^TM4^, and W161^EL2^, which facilitates cholesterol binding through hydrophobic interactions. Furthermore, the carbonyl oxygen of the CHS ester group is stabilized by a hydrogen bond with R195^EL2^ (Supplementary Fig. S9c). Cholesterol at the CHOL2 site is coordinated by residues Y128^TM3^, I203^EL2^, L206^EL2^, I435^TM9^, F438^TM9^, W534^TM10^, I538^TM10^, and L542^TM10^ (Supplementary Fig. S9b).

Previous studies have demonstrated that cholesterol can associate with transporters from the SLC6 family and modulate their function^48,49^. We compared two binding sites with the cholesterol-binding sites found in other homologous transporters. In the inward-facing structure of rGAT1^27^, no CHS or cholesterol molecule was determined at the corresponding CHOL1 site of BGT1, despite the residues within this pocket being almost conserved between BGT1 and GAT1, with the exception that S146 and R195 in BGT1 are replaced by N153 and N196 in GAT1, respectively (Supplementary Fig. S9d). Two cholesterol densities were observed around TM9 in GAT1 — one positioned within the groove formed by TM9, TM10, and TM12, which roughly overlaps the CHOL2 site found in BGT1. The second cholesterol-binding site is located close to TM9; however, there is no cholesterol binding at an equivalent position in BGT1, likely because F438 on TM9 of BGT1 prevents cholesterol from associating at this site (Supplementary Fig. S9e).

Additionally, we compared the cholesterol-binding site with those in the outward-open structure of hDAT^34^. Similar to rGAT1, we did not observe CHS binding at the CHOL1^BGT1^ site in the hDAT cryo-EM density map, probably because residue R195^BGT1^ was replaced (Supplementary Fig. S9f). These observations suggest that the interaction mediated by R195 in BGT1 is crucial for CHS binding. However, we detected one cholesterol molecule bound at an equivalent position of the CHOL2^BGT1^ site in DAT (Supplementary Fig. S9g). Considering that the structures of BGT1 and DAT are stabilized in inward- and outward-facing conformations, respectively, we speculate that cholesterol binding at CHOL2^BGT1^ is conformationally independent.

## Discussion

In this study, we determined high-resolution structures of WT BGT1, revealed the substrate binding and conformational transition mechanisms, and identified the Na3 site and two potential cholesterol-binding sites. Both endogenous substrates, GABA and betaine, bind in the same central cavity of BGT1 and exhibit nearly identical binding poses. The interactions involved in substrate binding are similar, but the water molecules that participate in substrate recognition differ. We propose that changes in the distribution of water molecules play a crucial thermodynamic role in accommodating different substrates in the binding pocket. BGT1 is capable of taking up betaine, unlike other transporters in the GAT family. Sequential and structural analyses have demonstrated that Q299 may be a key residue for the selectivity of BGT1 in transporting betaine. We hypothesize that the hydrophilicity of Q299, as opposed to the hydrophobic leucine in other transporters, creates a hydrophilic space in the substrate-binding pocket that is more favorable for betaine binding. Additionally, E52 plays an important role in betaine binding, with its side chain carboxyl group forming an electrostatic interaction with the positively charged quaternary amine of betaine, effectively stabilizing its binding. Notably, among GAT family members, E52 is highly conserved in BGT1, GAT2, and GAT3 but is replaced by Y60 in GAT1. Structural analysis revealed that Y60 in GAT1 can form a hydrogen bond with the amino group of GABA, thereby stabilizing substrate binding^26^, indicating that this site generally functions to stabilize substrate interactions.

Previous findings indicate that BGT1 plays a tissue-specific physiological role^50^, transporting predominantly GABA in the brain and betaine in the liver and kidney. However, the functions of these two substrates are not strictly segregated by tissue localization. For instance, under pathological conditions, glial cells may require betaine to regulate osmotic pressure^51^. Furthermore, BGT1 plays significant roles in hepatic apoptosis and inflammation, likely due to its role in GABA transport in the liver^52^. The similar binding modes of GABA and betaine indicate that substrate selectivity is not primarily determined by a distinct binding regulatory mechanism. Instead, BGT1 preference appears to be largely determined by substrate availability in specific tissues. High intracellular betaine concentrations in the kidney and liver favor betaine transport^53^, whereas in the brain, low betaine levels and abundant GABA promote GABA transport^54^. Together, these observations suggest that tissue-specific substrate availability underlies the distinct physiological roles of BGT1 across tissues.

BGT1 co-transports three sodium ions and one chloride ion per substrate, a ratio distinct from the 1:2:1 stoichiometry of substrate: Na^+^: Cl^–^ observed in other GAT proteins. We found that potential Na3 is coordinated by E93^TM2^, S104^IL1^, W108^IL1^, S122^TM2^, G300^TM6^, T303^TM6^, and E468^TM10^. Although S122 coordinates the Na^+^ ion indirectly through an intervening water molecule, it has a more pronounced impact on the function of the Na3 site than S104 does. This finding indicates that water-mediated coordination at a short distance is critical for maintaining Na3 binding and functional integrity. When we disrupted the putative Na3 site through mutation, we observed reverse transport as well as decreased saturated substrate accumulation, which is consistent with the role of BGT1 in the kidney, where it accumulates high concentrations of betaine to regulate osmotic pressure^55^. Disruption of the Na3 site may impair substrate transport by compromising conformational transitions and reducing the driving force for substrate translocation.

Our structural analysis of BGT1 revealed its occluded and inward-facing conformations. In the presence of R61^TM1^, Y64^TM1^, T289^TM6^, Q290^TM6^, D452^TM10^, and S457^TM10^, a hydrophilic pathway from the extracellular side to the substrate-binding pocket is blocked by a hydrogen bond network. E52^TM1^ and Q299^TM6^ interact with a water molecule, sealing the intracellular pathway and stabilizing the substrate in the binding pocket, resulting in the occluded state. In the GABA-bound structure, we capture the intermediate conformation between the occluded and inward-open states: TM1a tilts, and the side chain of E52^TM1^ shifts toward the cytoplasm, not fully opening a pathway to the intracellular side like it does in the inward-facing conformation of BGT1^inward-open^ (Supplementary Fig. S10).

In the BGT1 structure, we identified two potential cholesterol-binding sites, termed CHOL1 and CHOL2. Structural comparison revealed that the electrostatic interaction between R195 and the carboxyl group of CHS is critical for CHOL1 binding, suggesting that this site may not bind cholesterol under physiological conditions, as cholesterol lacks a carboxyl group. In contrast, CHOL2 is shared among SLC6 family members. Previous studies have highlighted the regulatory roles of cholesterol in transporters and the therapeutic potential of cholesterol-binding sites^49^. However, the cholesterol sites in BGT1 are located within scaffold helices rather than within helices directly involved in conformational transitions. These observations indicate that cholesterol binding at these sites may primarily contribute to structural stabilization rather than directly modulating transport.

In summary, we elucidate the structures of BGT1 in the occluded state bound with two distinct substrates and in the inward-facing conformation without exogenous substrate addition during purification, revealing the molecular basis underlying substrate recognition, conformational transitions, and ion coupling mechanisms, especially highlighting the physiological role of the Na3-binding site.

## Materials and methods

### Expression and purification of hBGT1

The full-length WT human BGT1 (*SLC6A12*, UniProt: P48065) was cloned and inserted into the pEG BacMam vector with a C-terminal fusion of the sfGFP fluorescent tag and the Twin-Strep tag. A PreScission protease site (LEVLFQ/GP) was inserted between BGT1 and the tags. The sequenced plasmids were subsequently transformed into DH10Bac *Escherichia coli* competent cells. White colonies were picked, and the bacmids were extracted to transfect sf9 cells, which were cultured in IB905 Medium (YSK BIOSCIENCES, Zhejiang, China) at 27 °C, yielding recombinant baculovirus. HEK293F cells at a concentration of ~2 × 10^6 ^cells/mL were infected with 1% (v/v) P2 virus and then cultured in 293F Hi-Exp medium (AC601501, Shanghai OPM Biosciences Co., Ltd) at 37 °C with 5% CO_2_. Sodium butyrate was added to the culture 24 h after transduction at a final concentration of 10 mM to promote protein expression. The cells were further cultured at 30 °C with 5% CO_2_ for 48 h. The cells were collected by centrifugation at 3000 rpm, immediately flash-frozen in liquid nitrogen, and stored at −80 °C for future use.

Cells were lysed in a Dounce homogenizer using basic buffer supplemented with 20 mM Tris, 150 mM NaCl (pH 8.0), 5 mM β-mercaptoethanol, and 10 mM GABA or 20 mM betaine. Protease inhibitors were added at final concentrations of 2 mg/mL aprotinin, 1.4 mg/mL leupeptin, and 0.5 mg/mL pepstatin A (MedChemExpress). The lysate was then ultracentrifuged at 35,000 rpm for 40 min at 4 °C, after which the cell membrane fraction was collected. The membrane fraction was solubilized at 4 °C for 2 h in the basic buffer supplemented with 1 mM ATP (Aladdin), 5 mM MgCl_2_, 1% (w/v) DDM, 0.15% (w/v) CHS, and protease inhibitors. Following solubilization, the sample was ultracentrifuged at 35,000 rpm for 40 min at 4 °C, and the supernatant was collected and filtered through a 0.22-μm filter. The filtered supernatant was then applied to a streptavidin affinity chromatography column (Smart-Life-sciences, China). The column was subsequently washed with the basic buffer supplemented with 1 mM ATP, 5 mM MgCl_2_, and 0.1% (w/v) digitonin, followed by the basic buffer supplemented with only 0.1% (w/v) digitonin. Elution was performed with the basic buffer supplemented with 0.1% (w/v) digitonin and 5 mM D-desthiobiotin (Cat# 1169249, Leyan, Shanghai, China). The eluate was concentrated and subjected to SEC using a Superose 6 Increase 10/300 GL column (GE Healthcare, USA) in the basic buffer supplemented with 0.1% (w/v) digitonin. The peak fractions were pooled and used for nanodisc reconstitution.

### Nanodisc reconstitution

Before reconstitution, brain polar lipid (BPL) was prepared as follows: 30 mg of lipid was dissolved in approximately 500 μL of chloroform and dried overnight under vacuum. The dried lipid film was then resuspended in buffer containing 20 mM HEPES and 150 mM KCl (pH 7.5) to a final concentration of 20 mg/mL. The suspension was thoroughly mixed by repeated passages (~10 times). Next, the lipid solution was sonicated for 15 min (with gentle inversion every 5 min to ensure homogeneity) and mixed using a 1 mL syringe. Afterward, the sample was subjected to three freeze‒thaw cycles in liquid nitrogen, followed by incubation in a 30 °C water bath for 5 min each time. Finally, the lipid suspension was extruded through a 0.4 μm polycarbonate membrane using a lipid extruder (~10 times) and stored in liquid nitrogen until further use.

Reconstitution was carried out using the protein: membrane scaffold protein MSP1D1E3: BPL at a molar ratio of 1:5:100. Lipids were perforated with 2% (w/v) digitonin for 30 min on ice before use. The lipids, MSP, and protein were mixed according to the specified ratio and incubated at 4 °C for 1 h. The Bio-Beads were weighed at a concentration of 600 mg/mL, and the mixture was added to the prepared Bio-Beads and incubated at 4 °C overnight to adsorb detergent. The reconstituted protein was then extracted from the Bio-Beads and centrifuged at 15,000 rpm to remove excess lipids. The supernatant was passed through a streptavidin affinity column to remove empty nanodiscs, after which it was washed with the basic purification buffer and eluted. PreScission protease was added to cleave the tags, and the mixture was re-applied to a Ni column to remove the His-tagged PreScission protease. The flow-through and wash fractions were collected, concentrated, and subjected to SEC. The peak fractions were collected. The resulting protein‒nanodisc reconstituted samples were concentrated to 7–10 mg/mL for cryo-EM sample preparation.

### Cryo-EM sample preparation and data acquisition

Quantifoil 1.2/1.3 Cu 300 mesh grids were glow-discharged for 60 s under H_2_–O_2_ conditions with a Solarus plasma cleaner (Gatan, USA) before 3 μL of protein sample was added to the grids and blotted using a Vitrobot Mark IV (Thermo Fisher Scientific, USA) (10 force, 4 s blotting time, and 100% humidity at 4 °C). The grids were then snap-frozen in liquid ethane and stored in liquid nitrogen before data acquisition. Cryo-EM images were collected on a Titan Krios G4 operated at 300 kV and equipped with a K3 Summit direct electron detector, using a slit width of 10 eV on a GIF-Quantum LS energy filter. Movie stacks were acquired using EPU software with a preset defocus ranging from −1.0 to −2.0 μm at a calibrated magnification of 105,000× in super-resolution mode. The pixel size on motion-corrected micrographs was 0.85 Å. Each movie stack was dose-fractioned in 32 frames. The dose rate was set to 15 e^–^/pixel/s, resulting in a total cumulative dose of 60 e^–^/Å^2^.

### Cryo-EM data processing

For BGT1^inward-open^, BGT1^GABA^, and BGT1^betaine^, 1681, 6066, and 2254 movie stacks were collected, respectively. The data processing workflow for the three samples was similar, and all the data were completed using cryoSPARC. Using BGT1^betaine^ as an example (Supplementary Fig. S2a), the detailed steps are as follows: the micrographs were motion corrected, followed by patch CTF estimation to determine the defocus value of each micrograph. Poor-quality and contaminated images were manually excluded. For the initial round of the BGT1^betaine^ dataset, 1,733,461 particles were picked up using a blob picker and extracted. After several rounds of 2D classification, we selected a subset of particles in which the transmembrane domain could be identified within the micelles. An ab initio model was generated using these 2D classes. After several rounds of heterogeneous refinement and non-uniform refinement, we obtained a 2.95 Å map with 301,917 particles as ‘seeds’^56^. From these particles, we manually selected ten classes with the clearest outlines and an average distribution of top and side views, from which we randomly selected 1000 particles for Topaz training. Afterward, we re-extracted the particles using Topaz extract, resulting in the generation of 1,413,046 particles. These particles were divided into classes with quantities similar to those of the ‘seeds’ and subjected to heterogeneous refinement with ‘seeds’ separately to recover any missed valuable particles for subsequent classification. After gathering and deduplicating all the selected particles, we obtained 689,889 high-quality particles and generated a 2.91 Å map. Subsequent 3D classification and local refinement were performed using a manually created mask, ultimately improving the resolution of BGT1^betaine^ to 2.60 Å.

### Model building and refinement

The initial model of BGT1^betaine^ was constructed manually based on the 2.6 Å cryo-EM map in COOT^57^. Structure refinement was performed using phenix.real_space_refine in PHENIX^58^, applying a secondary structure and Ramachandran restraints. For the ligands GABA and betaine, their structures and restraint files were generated using their PubChem Compound Identifiers through the Grade Web Server. These ligands were then docked into the map based on density. The modeling of BGT1^betaine^ was completed initially. Models of BGT1^GABA^ and BGT1^inward-open^ were subsequently built on this basis. The BGT1^betaine^ model was docked with the maps of BGT1^GABA^/BGT1^inward-open^ in UCSF Chimera^59^, followed by ligand replacement and refinement of structural details through COOT^57^ and PHENIX^60^. Structural validation statistics were derived from Phenix comprehensive validation (cryo-EM maps). Residues 170–180 of the extracellular loop 2, residues 588–614 at the C-terminus, and residues 1–42 at the N-terminus were missing in these models.

All figures were prepared using Open-Source PyMOL^61^ or UCSF Chimera^59^.

### [^3^H]GABA uptake assay

HEK293-T cells cultured in 24-well plates coated with poly-L-lysine (Sigma‒Aldrich) were used for transport measurements. The cells were transiently transfected with plasmids encoding BGT-WT and mutants using polyJeT (SignaGen) when the density reached approximately 70%–80%. Untransfected cells were used as blank controls. After 36 h, the DMEM was removed, and the cells were washed twice with 500 µL of pre-warmed assay buffer (HBSS supplemented with 20 mM HEPES, 1 mM CaCl_2_, and 1 mM MgCl_2_, pH 7.4). For uptake, the cells were incubated in 200 µL of pre-warmed assay buffer containing 5 nM [^3^H]GABA (75.3 Ci/mmol, 2.786 TBq/mmol, Revvity) for 3 min at 37 °C. Kinetics experiments were performed by incubating cells with assay buffer supplemented with GABA at different concentrations (0.1, 0.3, 1, 3, 10, 30, 100, 300 and 600 µM). The molar ratio of [^3^H]GABA to cold GABA was maintained at 1:100,000 for each measurement. The uptake was stopped by washing twice with 500 µL of ice-cold assay buffer. Afterward, the cells were lysed in 200 µL of assay buffer containing 1% Triton X-100 for 30 min at room temperature. A total of 160 µL of lysate was mixed with 1.5 mL of Ultima Gold XR LSC Cocktail (PerkinElmer) for scintillation counting using a Tri-Carb 5110TR Liquid Scintillation Counter (PerkinElmer).

To determine the effect of mutation on the amount of substrate accumulation in the cell, we performed saturation transport experiments. The cells were incubated with 200 µL of pre-warmed assay buffer containing 5 nM [^3^H]GABA for 3 min and 2 h at 37 °C. The 3-min unsaturated uptake assay was used as a positive control. After the incubation, the cells were washed and lysed to measure the radioactivity.

For the reverse transport experiments, 5 nM [^3^H]GABA was added to the assay buffer to initiate the uptake reaction. After being incubated for 3 min, the cells were immediately washed three times with 500 µL of ice-cold assay buffer. This moment was taken as the zero point of reverse transport, and the cells directly lysed to measure radioactivity were regarded as negative controls, and the reverse transport was assumed to be 0. The cells were then incubated with 200 µL of pre-warmed assay buffer for 30 min at 37 °C. The cells were subsequently washed twice and lysed as described above.

For experiments studying the dependence on external sodium, sodium was replaced by choline. The cells were subsequently washed twice with 500 µL of pre-warmed uptake buffer (0–300 mM choline Cl, 5 mM KCl, 1.2 mM MgSO_4_, 1.5 mM CaCl_2_, 1 mM KH_2_PO_4_, 5 mM glucose, 12.5 mM HEPES-K, pH 7.4). The cells were then incubated in 200 µL of pre-warmed uptake buffer containing 5 nM [^3^H]GABA and 500 µM GABA for 3 min at 37 °C. Uptake was stopped by washing twice with 500 µL of ice-cold uptake buffer, after which the samples were lysed to measure the radioactivity.

The expression levels of the mutants were determined by a surface biotinylation assay. The total protein concentration of the cells was determined using a BCA protein assay kit (Thermo Fisher Scientific). All the assays were repeated three times, and the data were analyzed with GraphPad Prism.

### Surface biotinylation assay

BGT1 WT and mutant constructs carrying a C-terminal FLAG tag were used for surface biotinylation. Sixty hours after baculovirus infection, 3.5 × 10^7^ 293F cells were collected and washed twice with HEPES buffer (20 mM HEPES, 150 mM NaCl, pH 7.5). The cells were then incubated at 4 °C for 1 h with gentle rotation in 1 mL of 1 mg/mL Sulfo-NHS-SS-Biotin to biotinylate the surface proteins. The reaction was quenched by washing the cells twice with HEPES buffer containing 10 mM glycine, followed by incubation at 4 °C for 20 min. The cell membranes were solubilized in lysis buffer at 4 °C for 1 h with gentle rotation, and the insoluble material was removed by centrifugation at 15,000× *g* for 30 min. The supernatant (800 μL) was incubated with 50 μL of streptavidin-conjugated resin under gravity flow. After extensive washing with wash buffer, the biotinylated proteins were eluted by incubating the resin with 50 μL loading buffer at room temperature for 30 min. The eluted proteins were separated by 12% SDS‒PAGE and transferred onto activated PVDF membranes (Yeasen Biotech) at 110 V for 60 min. The membranes were blocked with 5% milk in TBST at room temperature for 1 h and then incubated with primary antibodies for 1 h at room temperature. FLAG-tagged BGT1 proteins were detected using an anti-FLAG antibody, and Na^+^/K^+^-ATPase was used as a loading control. After being washed three times with TBST (5 min each), the membranes were incubated with HRP-conjugated secondary antibodies for 1 h at room temperature. Bands were visualized using enhanced chemiluminescence reagents.

## Supplementary information

Supplementary Information

## Data Availability

Three-dimensional cryo-EM density maps for BGT1^inward-open^, BGT1^betaine^, and BGT1^GABA^ have been deposited at the Electron Microscopy Data Bank (EMDB) under accession codes EMD-63238, EMD-63237, and EMD-63236, respectively. The coordinates for the BGT1^inward-open^, BGT1^betaine^, and BGT1^GABA^ models have been deposited at the Protein Data Bank (PDB) under accession codes 9LNO, 9LNN, and 9LNM, respectively. All the data required to support the conclusions of this study are provided within the main text and/or the Supplementary Information.
