# Supplementary material for: Structural basis of the transport mechanism of hBGT1
Source: Cell Discov. 2026 Sep 29;12:68. doi: 10.1038/s41421-026-00926-0 (PMC13620122; doi:10.1038/s41421-026-00926-0)
Supplement: Supplementary file 1 [file 41421_2026_926_MOESM1_ESM.pdf]

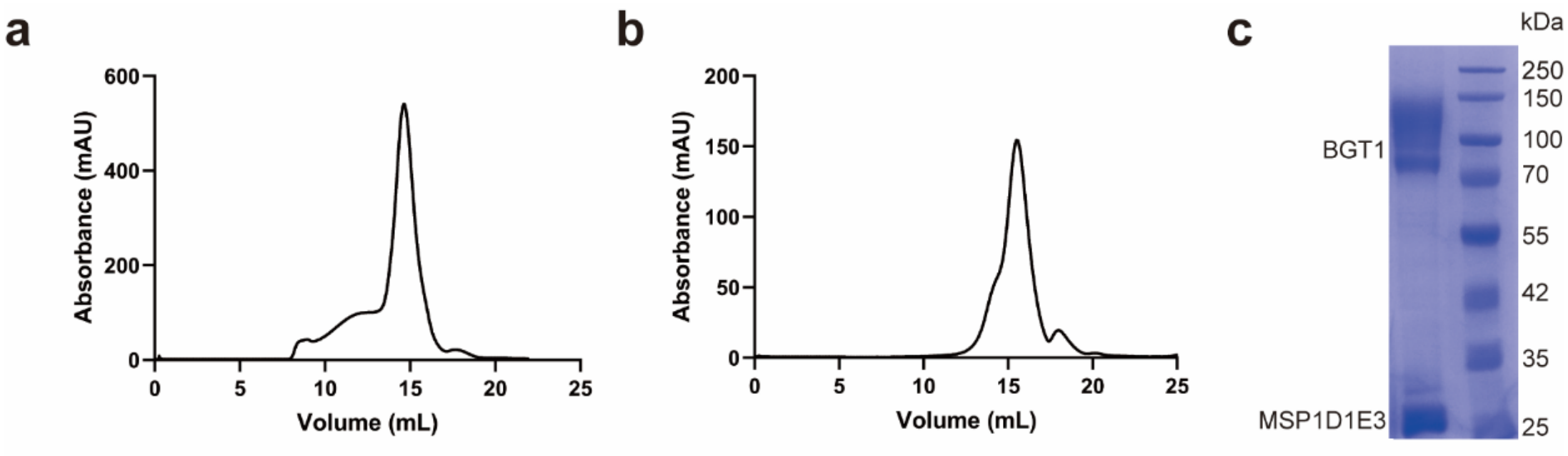


**Supplementary Fig. S1 Purification process of BGT1.**

(a) Representative SEC profile of BGT1-GFP in digitonin. Peak fractions were concentrated for nanodisc reconstitution. (b) Representative SEC profile of BGT1 reconstituted nanodisc. Peak fractions were concentrated for cryo-EM sample preparation. (c) SDS-PAGE gel of protein sample used to prepare cryo-EM sample, which is stained with Coomassie blue.

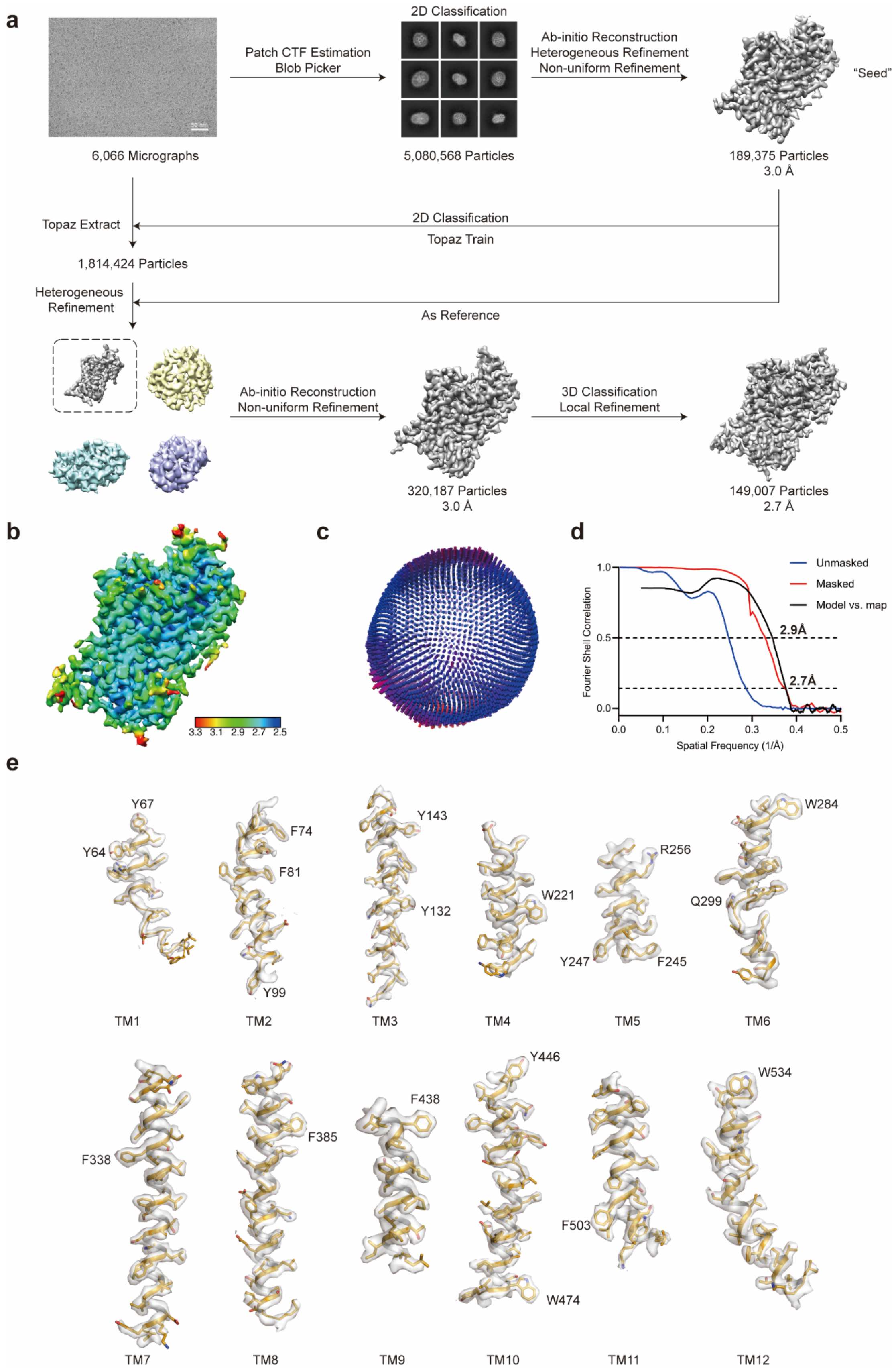


**Supplementary Fig. S2 Cryo-EM structural determination of BGT1 bound with GABA as a representative.**

(a)Workflow of data processing of BGT1 bound with GABA. See the Methods section for details. (b) Local resolution of the BGT1$^{GABA}$ cryo-EM density map. (c) Angular distribution of the 3D reconstruction for BGT1$^{GABA}$. (d) Fourier shell correlation (FSC) curves of model versus map (black), unmasked map (blue) and masked map (red). (e) Cryo-EM densities of transmembrane helices of BGT1$^{GABA}$ at a contour level of 4.5σ in PyMOL.

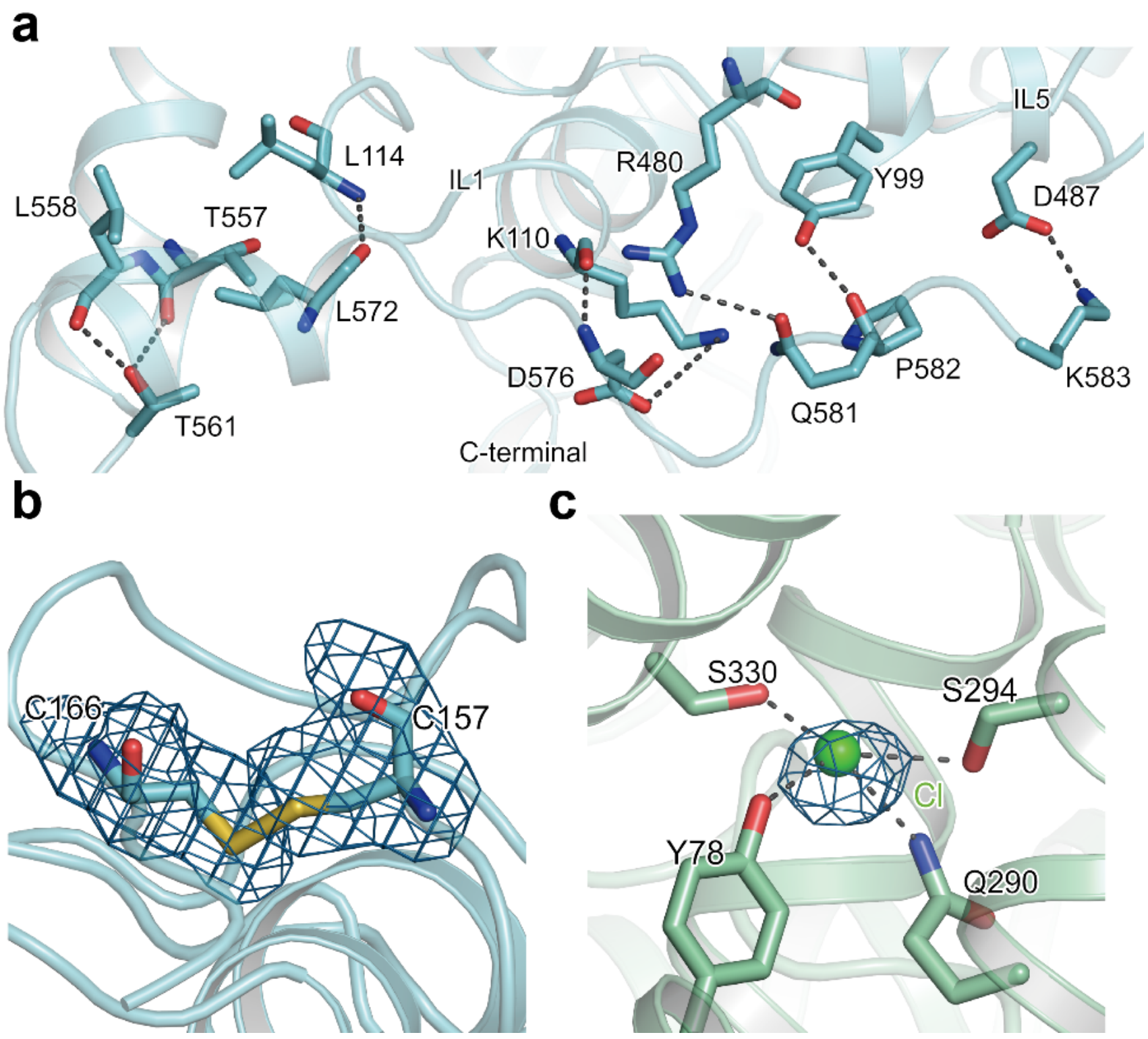


**Supplementary Fig. S3 Local details of BGT1 structure.**

(a) Interaction between C-terminal and nearby residues. Residues involved in coordination are depicted as sticks and labeled. Interactions are presented as black dashes. (b) Cryo-EM density of the disulfide bond formed by C157 and C166 at a contour level of 4σ in PyMOL. (c) Cryo-EM density of $Cl^-$ in structure of $BGT1^{inward\text{-}open}$ at a contour level of 4σ in PyMOL. Residues involved in interaction are shown as sticks and labeled. Coordination of $Cl^-$ is indicated with black dashes.

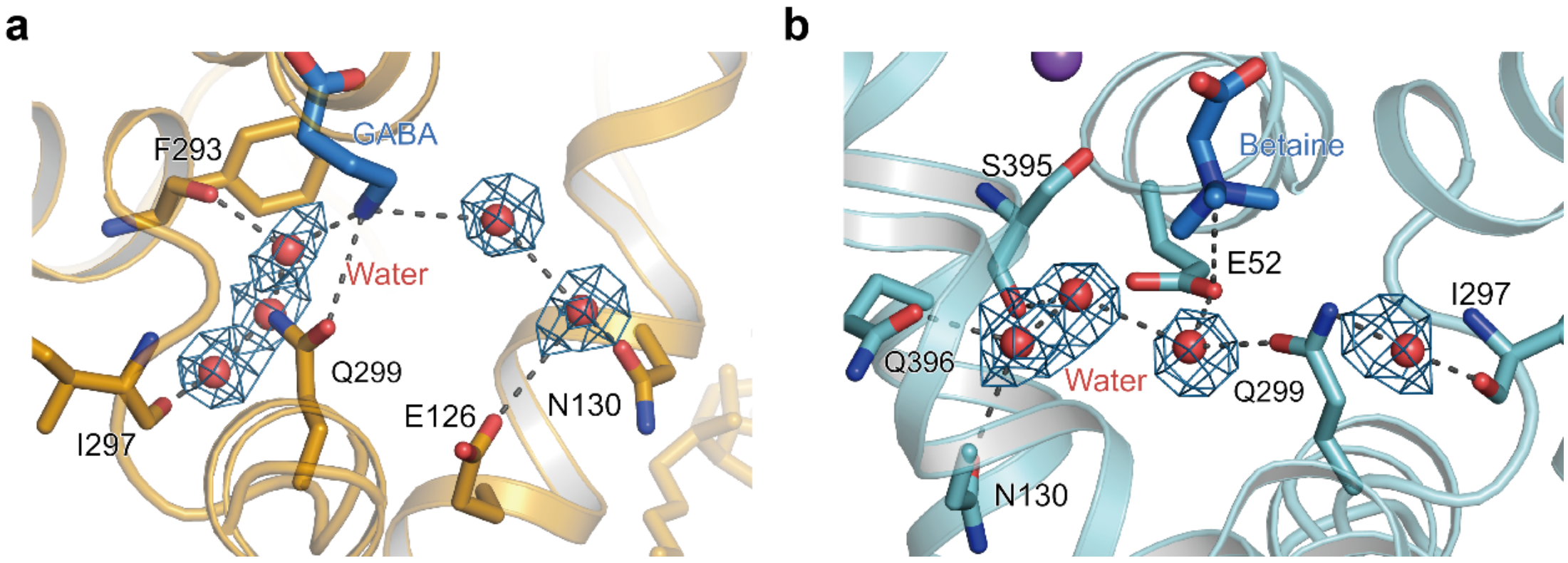


**Supplementary Fig. S4 Interaction of water molecules within the substrate binding pocket.**
(a) Hydrogen bonds formed by water molecules and residues in structure of $BGT1^{GABA}$. Water molecules are shown as red spheres. Residues involved in coordination are depicted as sticks and labeled. Possible hydrogen bonds are presented with black dashes. Cryo-EM density of water molecules is shown as mesh at a contour level of 3σ in PyMOL. (b) Hydrogen bonds formed by water molecules and residues in structure of $BGT1^{betaine}$. Representations are the same as (a). Cryo-EM density is contoured at 3σ in PyMOL.

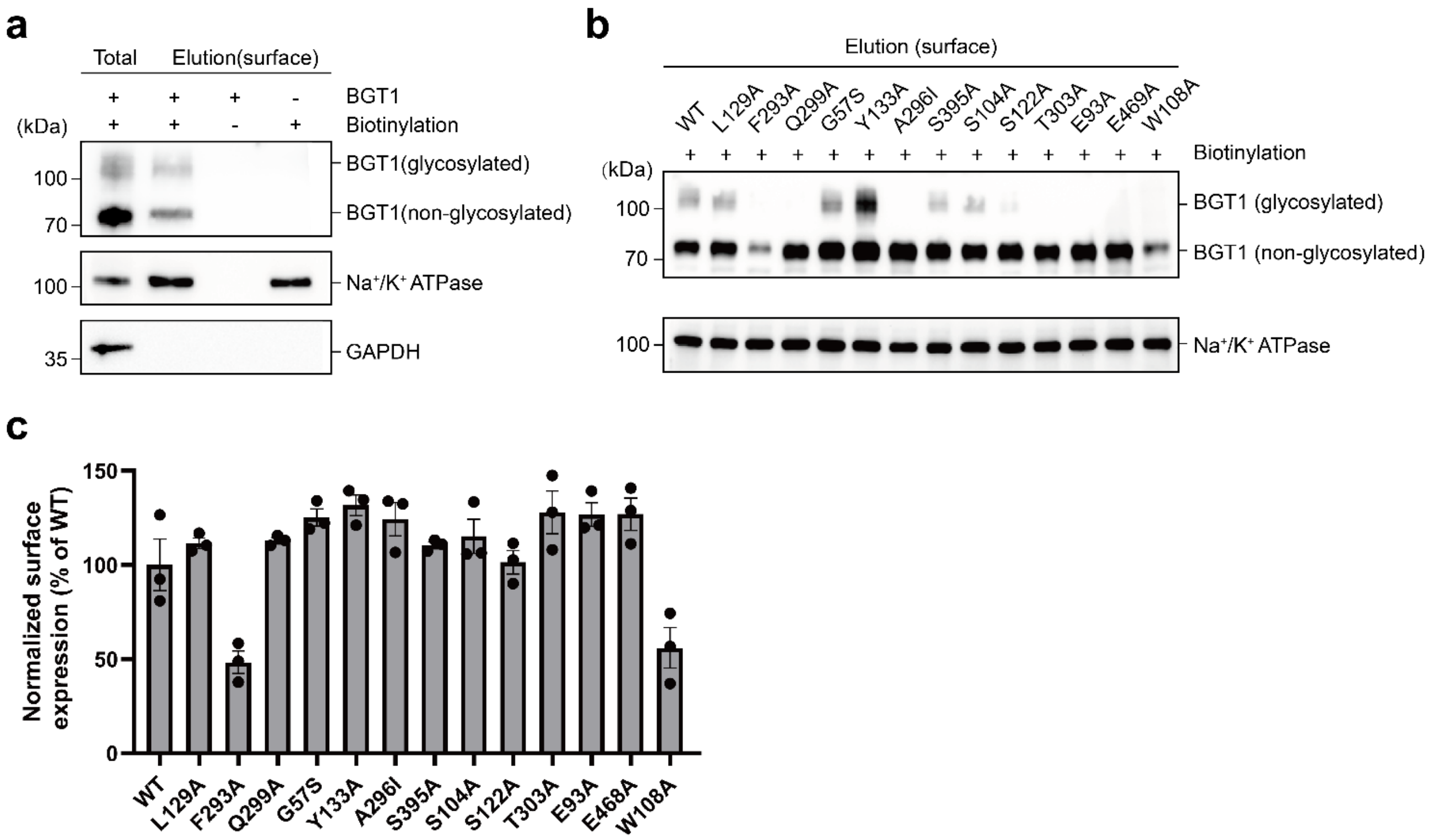


**Supplementary Fig. S5 Cell surface biotinylation analysis of $BGT1^{WT}$ and its mutants.**

(a) Cell surface biotinylation of $BGT1^{WT}$ in HEK293F cells. The samples of whole cell lysate and surface protein are labeled with biotin and recovered using streptavidin beads. No protein was detected in the elution from streptavidin beads for the unbiotinylated cell lysate sample. (b) Representative western blots of the surface $BGT1^{WT}$ and mutants. Biotinylated $Na^+/K^+$ ATPase on the cell surface was used as the loading control. (c) Densitometric quantification of surface expression for $BGT1^{WT}$ and its mutants. The gels derived from the same experiment were processed in parallel. The surface expression of wild-type and mutants was first normalized to $Na^+/K^+$ ATPase and then to the expression of wild-type. Each circular symbol represents an individual data point. Data are represented as mean ± S.E.M. n = 3 biologically independent experiments.

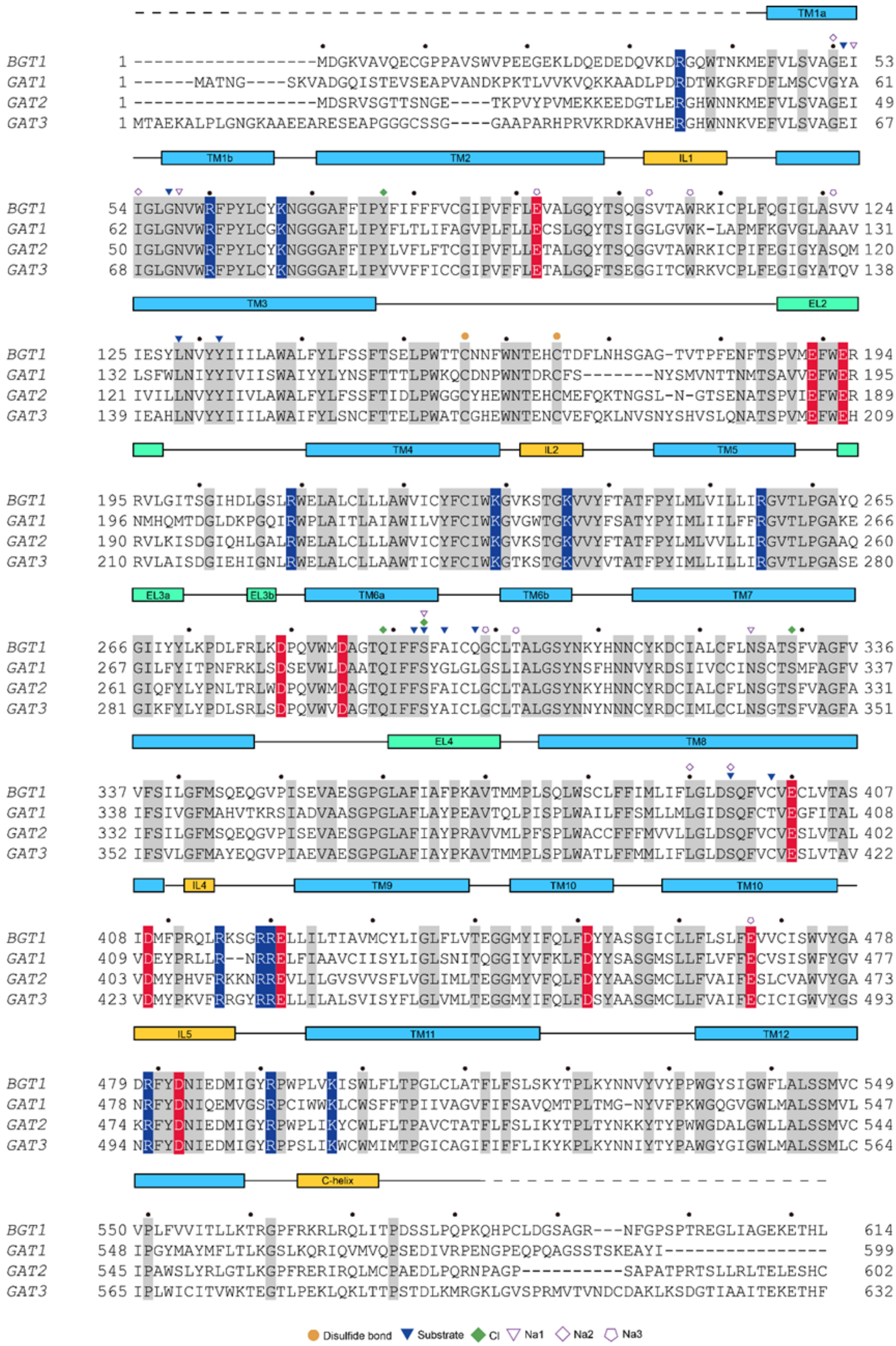


**Supplementary Fig. S6 Sequence alignment of GAT proteins.**

The four sequences are all from *Homo sapiens.* The conserved residues are shaded in gray, among which positively charged and negatively charged residues are highlighted in blue and red, respectively. The secondary elements of BGT1 are indicated above the sequences. Crucial residues involved in coordination of disulfide bond, substrate, Cl, Na1, Na2 and Na3 are indicated with orange circles, blue triangles, green

squares and purple hollow triangles, squares and pentagons, respectively.

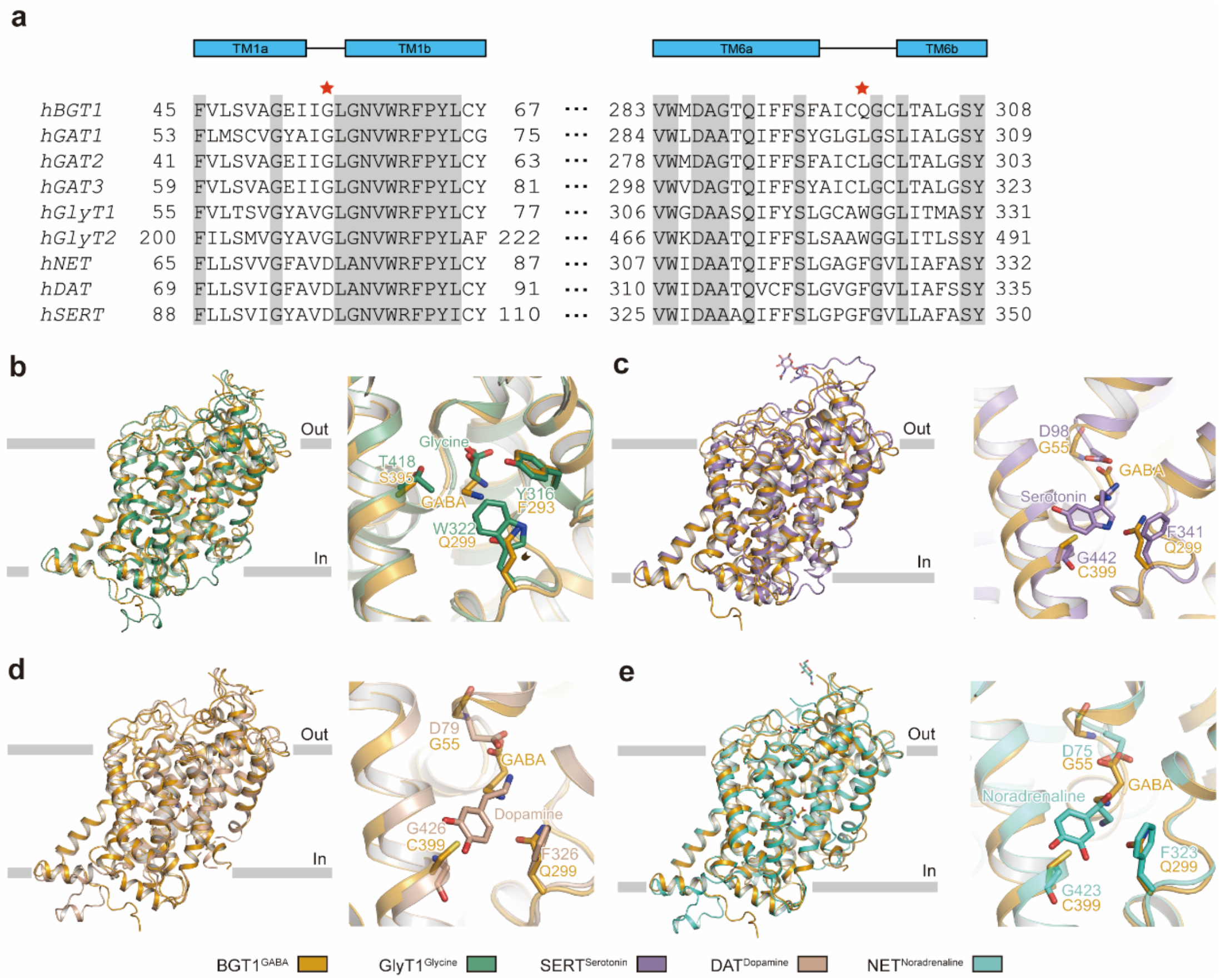


**Supplementary Fig. S7 Alignments of BGT1 with SLC6 proteins.**

(a) Sequence alignment of TM1 and TM6 of NTT proteins. G55$^{BGT1}$ and Q299$^{BGT1}$ are indicated with red stars. (b-e) Alignments of BGT1$^{GABA}$ with hGlyT1 (b, 8WFI), hSERT (c, 7MGW), hDAT (d, 8Y2D) and hNET (e, 8WTV) bound with corresponding substrates. Left are the overall alignments, while right are the alignments of substrate binding pockets.

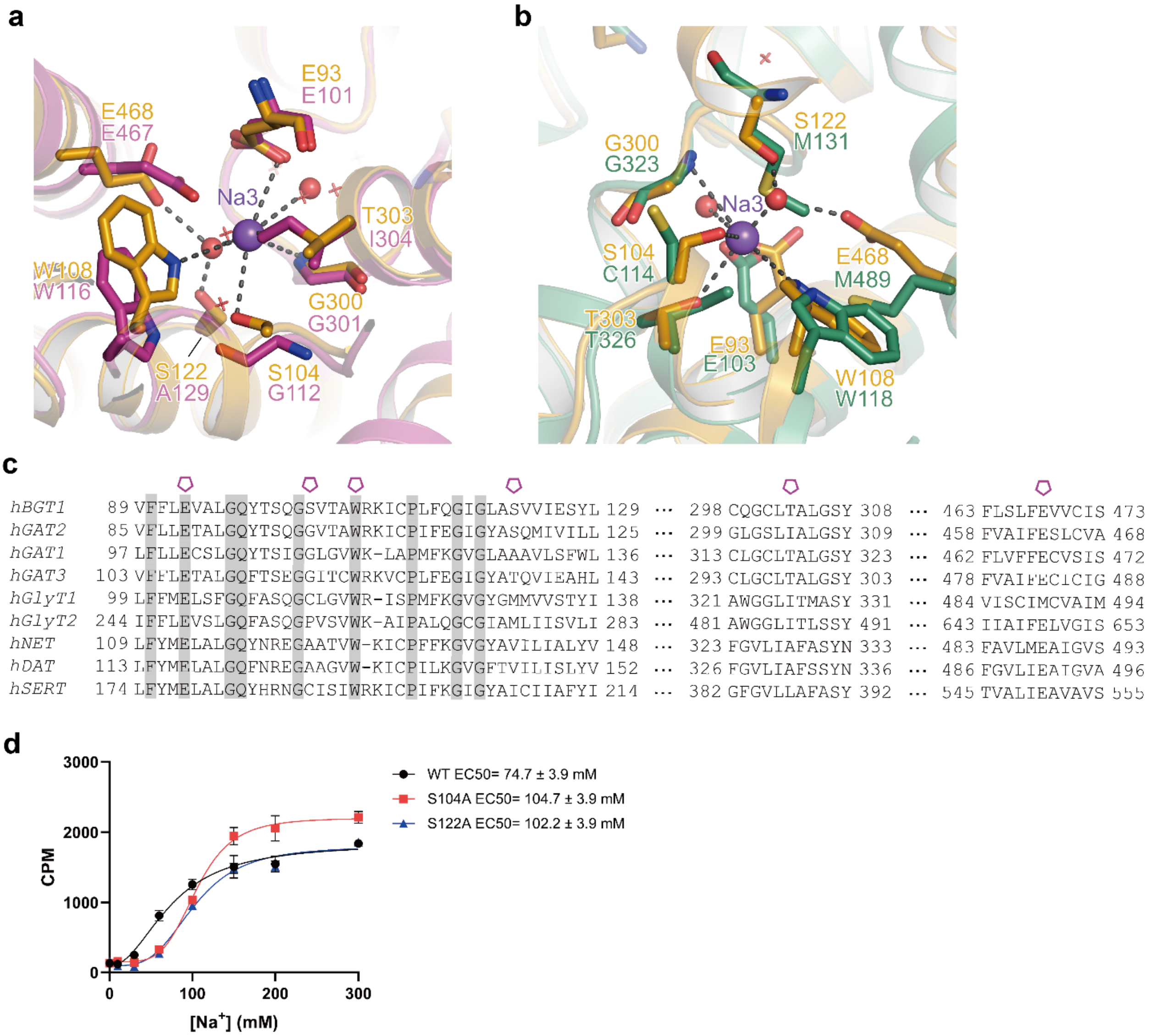


**Supplementary Fig. S8 Alignments of BGT1 Na3 site with hGAT1 and hGlyT1.**

(a, b) Structural comparison of Na3 site of BGT1 with hGAT1 (7Y7W) and hGlyT1 (8WFI), respectively. BGT1, GAT1, GlyT1 are shown in gold, purple and green, respectively. Crucial residues are labeled in corresponding colors. Interactions stabilizing $Na^+$ binding are represented as black dashes. Water molecules of hGAT1 are shown as red crosses. (c) Sequence alignment of Na3 site of NTT proteins. Crucial residues are indicated with purple hollow pentagons. (d) The $Na^+$-dependent substrate uptake kinetic curves for WT (black), S104A (red), and S122A (blue) of BGT1. Data were obtained in three biologically independent replicate experiments and fitted to the Hill equation. Data represent mean ± SEM (error bars), n = 3 biologically independent experiments. EC50 of WT, S122A, and S104A are 74.7 ± 3.9 mM, 104.7 ± 1.1 mM (p = 0.0020), and 102.2 ± 2.7 mM (p=0.0048), respectively. The Hill coefficients of WT, S104A and S122A were 2.0 ± 0.1, 4.8 ± 0.3 (p = 0.0006) and 3.5 ± 0.3 (p = 0.0053), respectively.

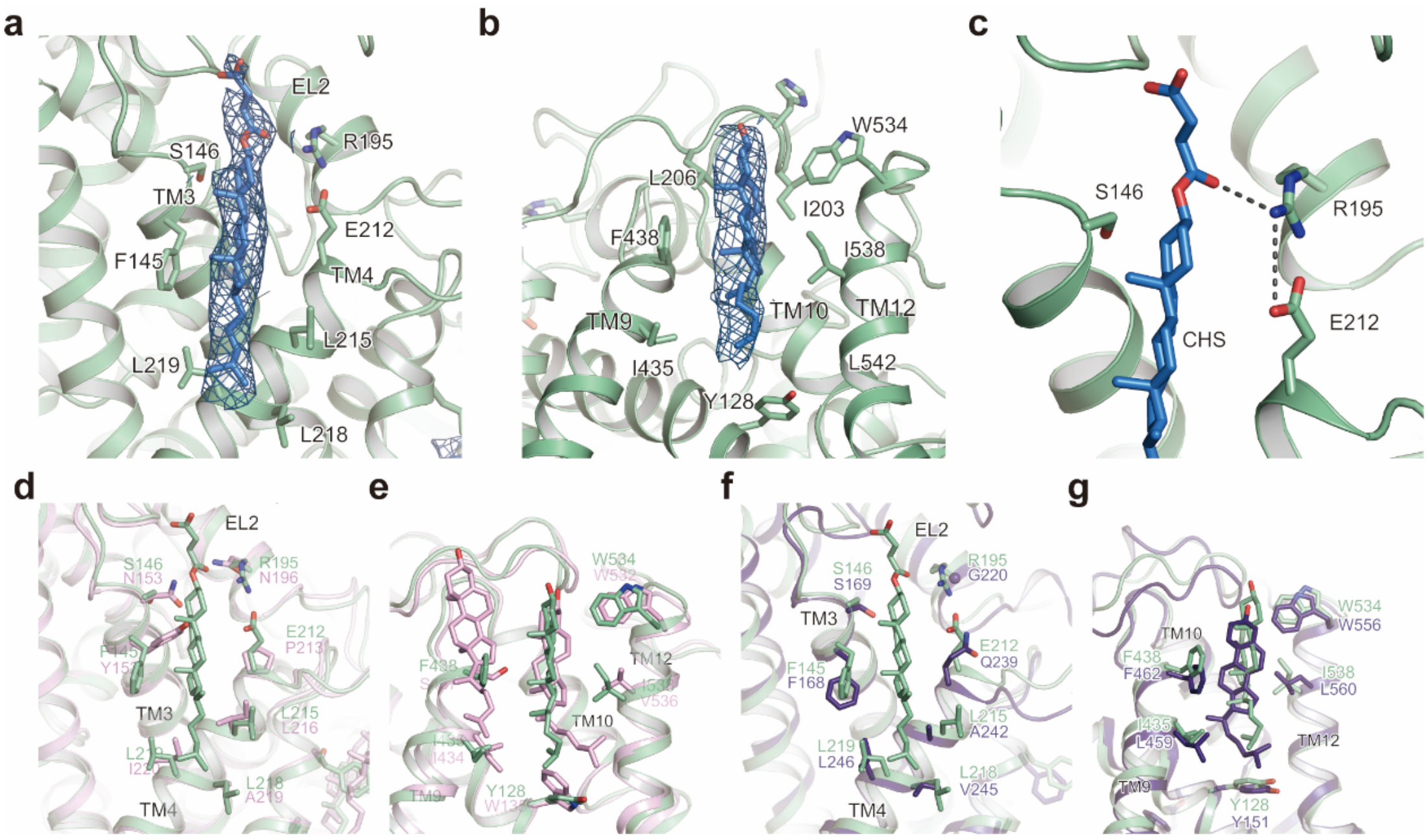


**Supplementary Fig. S9 Cryo-EM densities of lipid molecules.**

(a, b) Cryo-EM densities of lipid molecules located in CHOL1 (a) and CHOL2 (b) of BGT1$^{inward\text{-}open}$. Helices accommodating lipid molecules are labeled. Residues stabilizing the interaction are shown as sticks and labeled. Cryo-EM density is contoured at 5σ in PyMOL. (c) Interaction of hemisuccinate of CHS with BGT1. R195$^{EL2}$ forms electrostatic interaction with the carboxyl of CHS and E212$^{TM4}$. (d, e) Local structural alignment of CHOL1$^{BGT1}$ (d) and CHOL2$^{BGT1}$ (e) with rGAT1 (8GNK) (pink). BGT1$^{inward\text{-}open}$ is colored as green. Residues interacting with cholesterol molecules are shown as sticks and labeled with corresponding color. (f, g) Local structural alignment of CHOL1$^{BGT1}$ (f) and CHOL2$^{BGT1}$ (g) with hDAT (9EO4) (violet). Residues are shown as sticks and Cα of glycine is shown as sphere.

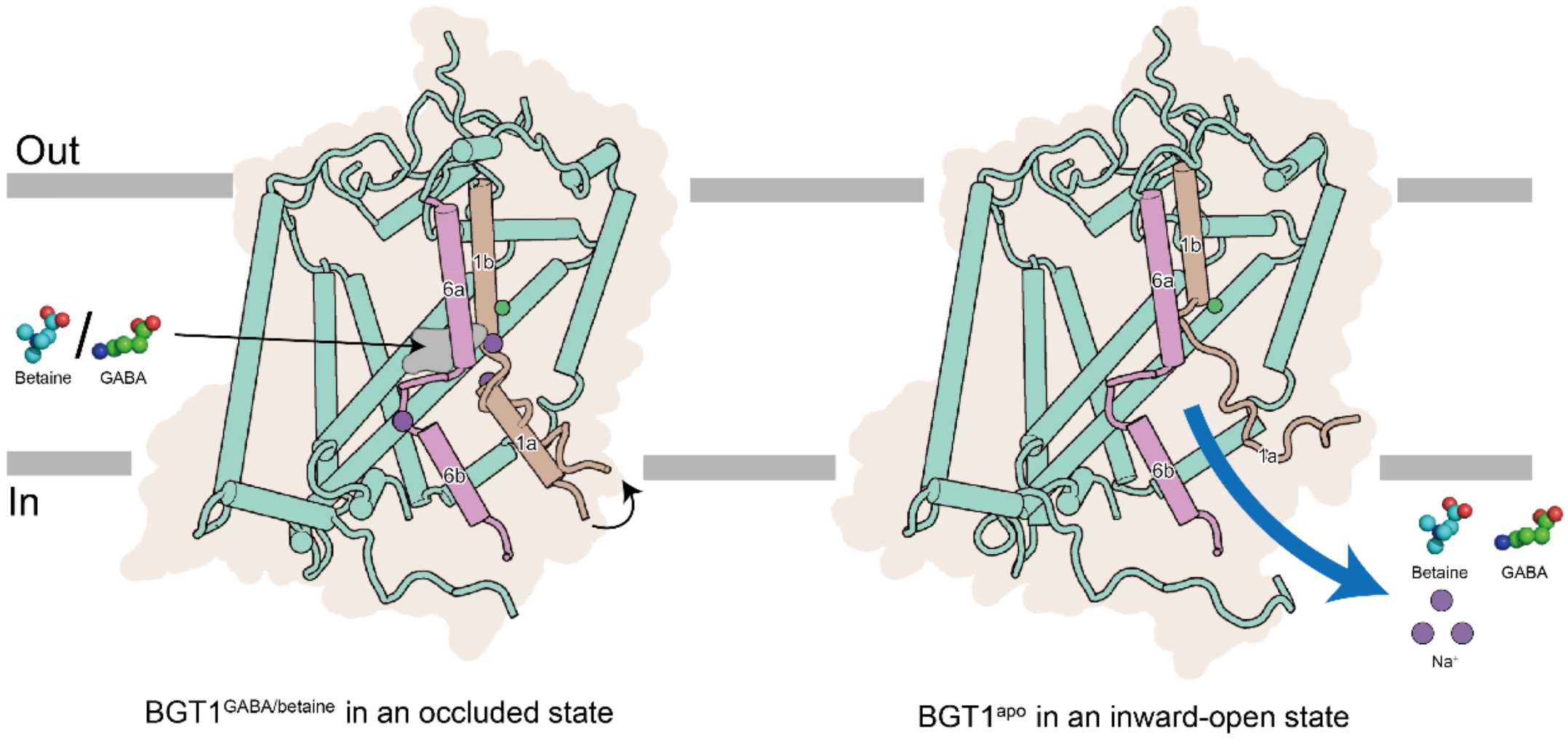


**Supplementary Fig. S10 Substrate transport mechanism of BGT1.**

The schematic illustrates the structural differences of BGT1 in the occluded and inward-open conformations. The gray area represents the substrate binding pocket. TM1 and TM6 are depicted in brown and magenta, respectively. The varying degrees of occluded conformations for GABA-bound and betaine-bound BGT1 are superposed, with the TM1a helix forming in betaine-bound BGT1, while it unwinds in GABA-bound BGT1. The black arrows indicate the direction of TM1a movement. Sodium ions and chloride ions are colored purple and green, respectively.

**Supplementary Table S1 Cryo-EM data collection, refinement and validation statistics**

| | BGT1$^{inward\text{-}open}$ (EMDB-63238) (PDB: 9LNO) | BGT1$^{GABA}$ (EMDB-63236) (PDB: 9LNM) | BGT1$^{betaine}$ (EMDB-63237) (PDB: 9LNN) |
|---|---|---|---|
| **Data collection and processing** | | | |
| Magnification | 105,000 × | 105,000 × | 105,000 × |
| Voltage (kV) | 300 | 300 | 300 |
| Electron exposure ($e^-$/Å$^2$) | 60 | 60 | 60 |
| Defocus range (μm) | −1.0 ~ −2.0 | −1.0 ~ −2.0 | −1.0 ~ −2.0 |
| Pixel size (Å) | 0.85 | 0.85 | 0.85 |
| Symmetry imposed | C1 | C1 | C1 |
| Initial particle images (no.) | 2,064,602 | 5,080,568 | 1,733,461 |
| Final particle images (no.) | 51,573 | 58,851 | 319,230 |
| Map resolution (Å) | 3.2 | 2.7 | 2.6 |
| FSC threshold | 0.143 | 0.143 | 0.143 |
| Map resolution range (Å) | 2.9 ~ 3.7 | 2.5 ~ 3.3 | 2.5 ~ 3.3 |
| **Refinement** | | | |
| Initial model used | BGT1$^{betaine}$ | BGT1$^{betaine}$ | *De novo* building |
| Model resolution (Å) | 3.5 | 2.9 | 2.8 |
| FSC threshold | 0.5 | 0.5 | 0.5 |
| Map sharpening *B* factor (Å$^2$) | 105.2 | 137.9 | 104.1 |
| **Model composition** | | | |
| Non-hydrogen atoms | 4248 | 4254 | 4231 |
| Protein residues | 520 | 519 | 519 |
| Ligands | 3 | 7 | 6 |
| *B* factors (Å$^2$) | | | |
| Protein | 82.61 | 40.24 | 33.91 |
| Ligand | 49.35 | 22.94 | 22.00 |
| R.m.s. deviations | | | |
| Bond lengths (Å) | 0.003 | 0.003 | 0.003 |
| Bond angles (°) | 0.595 | 0.609 | 0.598 |
| **Validation** | | | |
| MolProbity score | 1.48 | 1.30 | 1.19 |
| Clashscore | 4.01 | 3.75 | 2.97 |
| Poor rotamers (%) | 0.66 | 0.66 | 0.66 |
| CaBLAM outliers (%) | 0.98 | 0.79 | 0.99 |
| Ramachandran plot | | | |
| Favored (%) | 95.72 | 97.27 | 97.47 |
| Allowed (%) | 4.28 | 2.73 | 2.53 |
| Disallowed (%) | 0.00 | 0.00 | 0.00 |
